# Supplementary material for: HPA Axis Responsiveness Associates with Central Serotonin Transporter Availability in Human Obesity and Non-Obesity Controls
Source: Brain Sci. 2022 Oct 25;12(11):1430. doi: 10.3390/brainsci12111430 (PMC9688432; doi:10.3390/brainsci12111430)
Supplement: Supplementary file 1 [file brainsci-12-01430-s001.zip › 00_Open_Data_Description.pdf]

Open Data

"HPA axis responsiveness associates with central serotonin transporter availability in human obesity and non-obesity controls" Schinke et al.

List of content

00_Open_Data_Description.docx

01_Data_public.sav

02_Syntax.sps

03_Statistics_Output.spv

03b_Statistics_Output.pdf

04_Fig_1_and_Suppl_Fig_1.pzf

05_Fig_2-5_Suppl_Fig_2-4.pzfx

Description

01_Data_public.docx: This file contains the SPSS table with the original data (participant characteristics, dex/CRH test results, 5-HTT BPND of dedicated brain areas).

02_Syntax.sav This file provides the syntax with which the results were calculated. It can be run to create the results which are given in 03_Statistics_Output.spv.

03_Statistics_Output.spv. All data stated in the manuscript are given here.

03b_Statistics_Output.pdf. This is the corresponding PDF file to 03_Statistics_Output.spv.

04_Fig_1_and_Suppl_Fig_1.pzf. This is the GraphPad Prism file which contains the Figure 1 and the Supplementary Figure 1. By clicking on the respective file in the *Layout section,* the figure panels and its calculation can be found.

05_Fig_2-5_Suppl_Fig_2-4.pzfx. This is the GraphPad Prism file which contains the Figures 2-5 and the Supplementary Figure 2-4. By clicking on the respective file in the *Layout section,* the figure panels and its calculation can be found.
