# Supplementary material for: HPA Axis Responsiveness Associates with Central Serotonin Transporter Availability in Human Obesity and Non-Obesity Controls
Source: Brain Sci. 2022 Oct 25;12(11):1430. doi: 10.3390/brainsci12111430 (PMC9688432; doi:10.3390/brainsci12111430)
Supplement: Supplementary file 1 [file brainsci-12-01430-s001.zip › 03b_Statistics_Output.pdf]

Explore

Notes

Output Created		21-SEP-2022 00:01:04
Comments		
Input	Data	C:\Users\Christian Schinke\OneDrive - Charité - Universitätsmedizin Berlin\Promotion und Projekte Leipzig\02_DASB vs. HPA\06_Open_Data\01_Data_public.sav
	Active Dataset	DataSet1
	Filter	NOT INCLUSION_EXCLUSION =9 (FILTER)
	Weight	<none>
	Split File	Status (0=NC,1=ADI)
	N of Rows in Working Data File	40
Missing Value Handling	Definition of Missing	User-defined missing values for dependent variables are treated as missing.
	Cases Used	Statistics are based on cases with no missing values for the dependent variable or factor(s) being analyzed.

Notes

Syntax		EXAMINE VARIABLES=Age BMI_Baseline_PET BDI SCL_90_tANX BAS_Drive BAS_FUN BAS_Reward BIS ACTH1500<br>ACTH1530_postCRH ACTH1545 ACTH1600 ACTH1615 CRT1500 CRT1530_postCRH CRT1545 CRT1600 CRT1615<br>ACTH_MAX ACTH_dMAX ACTH_AUC CRT_MAX CRT_dMAX CRT_AUC AC_ratio_postCRH_1530 AC_ratio_MAX<br>AC_ratio_AUC SERT_BPND_Group_average FC OFC DLPFC ACC INS HI AMG NAcc CD PU TH HYPOTH SN_VTA MIB<br>PONS<br>/PLOT BOXPLOT NPPLOT<br>/COMPARE GROUPS<br>/STATISTICS DESCRIPTIVES<br>/CINTERVAL 95<br>/MISSING PAIRWISE...
Resources	Processor Time	00:00:20,50
	Elapsed Time	00:00:18,62

Case Processing Summary

	Status (0=NC,1=ADI)					
	LC					
	Cases					
	Valid		Missing		Total	
	N	Percent	N	Percent	N	Percent
Age-BS	12	100,0%	0	0,0%	12	100,0%
BMI-BS	12	100,0%	0	0,0%	12	100,0%
Beck Depression Inventory	12	100,0%	0	0,0%	12	100,0%
SCL_90_tANX	12	100,0%	0	0,0%	12	100,0%
BAS_Drive	12	100,0%	0	0,0%	12	100,0%
BAS_FUN	12	100,0%	0	0,0%	12	100,0%
BAS_Reward	12	100,0%	0	0,0%	12	100,0%
BIS	12	100,0%	0	0,0%	12	100,0%
ACTH1500	12	100,0%	0	0,0%	12	100,0%
ACTH1530_postCRH	12	100,0%	0	0,0%	12	100,0%
ACTH1545	12	100,0%	0	0,0%	12	100,0%
ACTH1600	12	100,0%	0	0,0%	12	100,0%
ACTH1615	12	100,0%	0	0,0%	12	100,0%
CRT1500	12	100,0%	0	0,0%	12	100,0%
CRT1530_postCRH	12	100,0%	0	0,0%	12	100,0%
CRT1545	12	100,0%	0	0,0%	12	100,0%
CRT1600	12	100,0%	0	0,0%	12	100,0%
CRT1615	12	100,0%	0	0,0%	12	100,0%
Stress-ACTH-max	12	100,0%	0	0,0%	12	100,0%
Stress-ACTH-delta-max	12	100,0%	0	0,0%	12	100,0%
Stress-A-AUC	12	100,0%	0	0,0%	12	100,0%
Stress-C-max	12	100,0%	0	0,0%	12	100,0%
Stress-C-dmax	12	100,0%	0	0,0%	12	100,0%
Stress-C-AUC	12	100,0%	0	0,0%	12	100,0%
AC_ratio_postCRH_1530	12	100,0%	0	0,0%	12	100,0%
AC_ratio_MAX	12	100,0%	0	0,0%	12	100,0%
AC_ratio_AUC	12	100,0%	0	0,0%	12	100,0%
VOI-BS_NUK_Group	12	100,0%	0	0,0%	12	100,0%
Frontal cortex	12	100,0%	0	0,0%	12	100,0%
Orbitofronta cortex	12	100,0%	0	0,0%	12	100,0%
Dorsolateral prefrontal cortex	12	100,0%	0	0,0%	12	100,0%
Anterior cingulate cortex	12	100,0%	0	0,0%	12	100,0%

Case Processing Summary

	Status (0=NC,1=ADI)					
	OB					
	Cases					
	Valid		Missing		Total	
	N	Percent	N	Percent	N	Percent
Age-BS	28	100,0%	0	0,0%	28	100,0%
BMI-BS	28	100,0%	0	0,0%	28	100,0%
Beck Depression Inventory	28	100,0%	0	0,0%	28	100,0%
SCL_90_tANX	20	71,4%	8	28,6%	28	100,0%
BAS_Drive	28	100,0%	0	0,0%	28	100,0%
BAS_FUN	28	100,0%	0	0,0%	28	100,0%
BAS_Reward	28	100,0%	0	0,0%	28	100,0%
BIS	28	100,0%	0	0,0%	28	100,0%
ACTH1500	28	100,0%	0	0,0%	28	100,0%
ACTH1530_postCRH	28	100,0%	0	0,0%	28	100,0%
ACTH1545	28	100,0%	0	0,0%	28	100,0%
ACTH1600	28	100,0%	0	0,0%	28	100,0%
ACTH1615	28	100,0%	0	0,0%	28	100,0%
CRT1500	28	100,0%	0	0,0%	28	100,0%
CRT1530_postCRH	28	100,0%	0	0,0%	28	100,0%
CRT1545	28	100,0%	0	0,0%	28	100,0%
CRT1600	28	100,0%	0	0,0%	28	100,0%
CRT1615	28	100,0%	0	0,0%	28	100,0%
Stress-ACTH-max	28	100,0%	0	0,0%	28	100,0%
Stress-ACTH-delta-max	28	100,0%	0	0,0%	28	100,0%
Stress-A-AUC	28	100,0%	0	0,0%	28	100,0%
Stress-C-max	28	100,0%	0	0,0%	28	100,0%
Stress-C-dmax	28	100,0%	0	0,0%	28	100,0%
Stress-C-AUC	28	100,0%	0	0,0%	28	100,0%
AC_ratio_postCRH_1530	28	100,0%	0	0,0%	28	100,0%
AC_ratio_MAX	28	100,0%	0	0,0%	28	100,0%
AC_ratio_AUC	28	100,0%	0	0,0%	28	100,0%
VOI-BS_NUK_Group	28	100,0%	0	0,0%	28	100,0%
Frontal cortex	28	100,0%	0	0,0%	28	100,0%
Orbitofronta cortex	28	100,0%	0	0,0%	28	100,0%
Dorsolateral prefrontal cortex	28	100,0%	0	0,0%	28	100,0%
Anterior cingulate cortex	28	100,0%	0	0,0%	28	100,0%

Case Processing Summary

	Status (0=NC,1=ADI) LC					
	Cases					
	Valid		Missing		Total	
	N	Percent	N	Percent	N	Percent
Insula	12	100,0%	0	0,0%	12	100,0%
Hippocampus	12	100,0%	0	0,0%	12	100,0%
Amygdala	12	100,0%	0	0,0%	12	100,0%
Ncl. accumbens	12	100,0%	0	0,0%	12	100,0%
Head of the caudate	12	100,0%	0	0,0%	12	100,0%
Putamen	12	100,0%	0	0,0%	12	100,0%
Thalamus	12	100,0%	0	0,0%	12	100,0%
Hypothalamus	12	100,0%	0	0,0%	12	100,0%
Substantia nigra	12	100,0%	0	0,0%	12	100,0%
Midbrain	12	100,0%	0	0,0%	12	100,0%
Pons	12	100,0%	0	0,0%	12	100,0%

Case Processing Summary

	Status (0=NC,1=ADI) OB					
	Cases					
	Valid		Missing		Total	
	N	Percent	N	Percent	N	Percent
Insula	28	100,0%	0	0,0%	28	100,0%
Hippocampus	28	100,0%	0	0,0%	28	100,0%
Amygdala	28	100,0%	0	0,0%	28	100,0%
Ncl. accumbens	28	100,0%	0	0,0%	28	100,0%
Head of the caudate	28	100,0%	0	0,0%	28	100,0%
Putamen	28	100,0%	0	0,0%	28	100,0%
Thalamus	28	100,0%	0	0,0%	28	100,0%
Hypothalamus	28	100,0%	0	0,0%	28	100,0%
Substantia nigra	28	100,0%	0	0,0%	28	100,0%
Midbrain	28	100,0%	0	0,0%	28	100,0%
Pons	28	100,0%	0	0,0%	28	100,0%

Descriptives

				Status (0=NC,1=ADI)
				LC
Age-BS	Mean		Statistic	35,75
			Std. Error	2,125
	95% Confidence Interval for Mean	Lower Bound	Statistic	31,07
		Upper Bound	Statistic	40,43
	5% Trimmed Mean		Statistic	35,83
	Median		Statistic	36,00
	Variance		Statistic	54,205
	Std. Deviation		Statistic	7,362
	Minimum		Statistic	21
	Maximum		Statistic	49
	Range		Statistic	28
	Interquartile Range		Statistic	10
	Skewness		Statistic	-,285
			Std. Error	,637
	Kurtosis		Statistic	,521
			Std. Error	1,232
BMI-BS	Mean		Statistic	22,401
			Std. Error	,6537
	95% Confidence Interval for Mean	Lower Bound	Statistic	20,962
		Upper Bound	Statistic	23,840
	5% Trimmed Mean		Statistic	22,367
	Median		Statistic	22,440
	Variance		Statistic	5,128
	Std. Deviation		Statistic	2,2646
	Minimum		Statistic	19,8
	Maximum		Statistic	25,6
	Range		Statistic	5,7
	Interquartile Range		Statistic	4,6
	Skewness		Statistic	,082
			Std. Error	,637
	Kurtosis		Statistic	-1,919
			Std. Error	1,232
Beck Depression Inventory	Mean		Statistic	1,1667
			Std. Error	,56183
	95% Confidence Interval for	Lower Bound	Statistic	-,0699

Descriptives

				Status (0=NC,1=ADI)
				OB
Age-BS	Mean		Statistic	36,57
			Std. Error	1,998
	95% Confidence Interval for Mean	Lower Bound	Statistic	32,47
		Upper Bound	Statistic	40,67
	5% Trimmed Mean		Statistic	36,22
	Median		Statistic	35,00
	Variance		Statistic	111,735
	Std. Deviation		Statistic	10,570
	Minimum		Statistic	21
	Maximum		Statistic	59
	Range		Statistic	38
	Interquartile Range		Statistic	15
	Skewness		Statistic	,486
			Std. Error	,441
	Kurtosis		Statistic	-,549
			Std. Error	,858
BMI-BS	Mean		Statistic	41,152
			Std. Error	,9620
	95% Confidence Interval for Mean	Lower Bound	Statistic	39,178
		Upper Bound	Statistic	43,126
	5% Trimmed Mean		Statistic	40,809
	Median		Statistic	39,801
	Variance		Statistic	25,913
	Std. Deviation		Statistic	5,0905
	Minimum		Statistic	35,5
	Maximum		Statistic	54,1
	Range		Statistic	18,6
	Interquartile Range		Statistic	8,0
	Skewness		Statistic	,801
			Std. Error	,441
	Kurtosis		Statistic	-,104
			Std. Error	,858
Beck Depression Inventory	Mean		Statistic	7,2500
			Std. Error	,92956
	95% Confidence Interval for	Lower Bound	Statistic	5,3427

Descriptives

				Status (0=NC,1=ADI)
				LC
	Mean	Upper Bound	Statistic	2,4033
	5% Trimmed Mean		Statistic	1,0185
	Median		Statistic	,0000
	Variance		Statistic	3,788
	Std. Deviation		Statistic	1,94625
	Minimum		Statistic	,00
	Maximum		Statistic	5,00
	Range		Statistic	5,00
	Interquartile Range		Statistic	3,25
	Skewness		Statistic	1,319
			Std. Error	,637
	Kurtosis		Statistic	-,084
			Std. Error	1,232
SCL_90_tANX	Mean		Statistic	44,7500
			Std. Error	1,64282
	95% Confidence Interval for Mean	Lower Bound	Statistic	41,1342
		Upper Bound	Statistic	48,3658
	5% Trimmed Mean		Statistic	44,7222
	Median		Statistic	45,0000
	Variance		Statistic	32,386
	Std. Deviation		Statistic	5,69090
	Minimum		Statistic	38,00
	Maximum		Statistic	52,00
	Range		Statistic	14,00
	Interquartile Range		Statistic	10,25
	Skewness		Statistic	-,007
			Std. Error	,637
	Kurtosis		Statistic	-1,982
			Std. Error	1,232
BAS_Drive	Mean		Statistic	13,1667
			Std. Error	,36584
	95% Confidence Interval for	Lower Bound	Statistic	12,3615

Descriptives

				Status (0=NC,1=ADI)
				OB
	Mean	Upper Bound	Statistic	9,1573
	5% Trimmed Mean		Statistic	7,1429
	Median		Statistic	6,5000
	Variance		Statistic	24,194
	Std. Deviation		Statistic	4,91878
	Minimum		Statistic	,00
	Maximum		Statistic	17,00
	Range		Statistic	17,00
	Interquartile Range		Statistic	8,00
	Skewness		Statistic	,244
			Std. Error	,441
	Kurtosis		Statistic	-,958
			Std. Error	,858
SCL_90_tANX	Mean		Statistic	48,9500
			Std. Error	1,69748
	95% Confidence Interval for Mean	Lower Bound	Statistic	45,3971
		Upper Bound	Statistic	52,5029
	5% Trimmed Mean		Statistic	48,5000
	Median		Statistic	49,0000
	Variance		Statistic	57,629
	Std. Deviation		Statistic	7,59137
	Minimum		Statistic	38,00
	Maximum		Statistic	68,00
	Range		Statistic	30,00
	Interquartile Range		Statistic	11,00
	Skewness		Statistic	,708
			Std. Error	,512
	Kurtosis		Statistic	,609
			Std. Error	,992
BAS_Drive	Mean		Statistic	12,5000
			Std. Error	,39675
	95% Confidence Interval for	Lower Bound	Statistic	11,6859

Descriptives

				Status (0=NC,1=ADI)
				LC
	Mean	Upper Bound	Statistic	13,9719
	5% Trimmed Mean		Statistic	13,1852
	Median		Statistic	14,0000
	Variance		Statistic	1,606
	Std. Deviation		Statistic	1,26730
	Minimum		Statistic	11,00
	Maximum		Statistic	15,00
	Range		Statistic	4,00
	Interquartile Range		Statistic	2,00
	Skewness		Statistic	-,369
			Std. Error	,637
	Kurtosis		Statistic	-1,367
			Std. Error	1,232
BAS_FUN	Mean		Statistic	12,4167
			Std. Error	,48396
	95% Confidence Interval for Mean	Lower Bound	Statistic	11,3515
		Upper Bound	Statistic	13,4819
	5% Trimmed Mean		Statistic	12,4630
	Median		Statistic	12,5000
	Variance		Statistic	2,811
	Std. Deviation		Statistic	1,67649
	Minimum		Statistic	9,00
	Maximum		Statistic	15,00
	Range		Statistic	6,00
	Interquartile Range		Statistic	1,75
	Skewness		Statistic	-,660
			Std. Error	,637
	Kurtosis		Statistic	,514
			Std. Error	1,232
BAS_Reward	Mean		Statistic	17,25
			Std. Error	,538
	95% Confidence Interval for	Lower Bound	Statistic	16,07

Descriptives

				Status (0=NC,1=ADI)
				OB
	Mean	Upper Bound	Statistic	13,3141
	5% Trimmed Mean		Statistic	12,6349
	Median		Statistic	13,0000
	Variance		Statistic	4,407
	Std. Deviation		Statistic	2,09938
	Minimum		Statistic	7,00
	Maximum		Statistic	15,00
	Range		Statistic	8,00
	Interquartile Range		Statistic	2,75
	Skewness		Statistic	-,828
			Std. Error	,441
	Kurtosis		Statistic	,279
			Std. Error	,858
BAS_FUN	Mean		Statistic	11,0714
			Std. Error	,37420
	95% Confidence Interval for Mean	Lower Bound	Statistic	10,3036
		Upper Bound	Statistic	11,8392
	5% Trimmed Mean		Statistic	10,9841
	Median		Statistic	11,0000
	Variance		Statistic	3,921
	Std. Deviation		Statistic	1,98006
	Minimum		Statistic	8,00
	Maximum		Statistic	16,00
	Range		Statistic	8,00
	Interquartile Range		Statistic	2,00
	Skewness		Statistic	,634
			Std. Error	,441
	Kurtosis		Statistic	,234
			Std. Error	,858
BAS_Reward	Mean		Statistic	16,31
			Std. Error	,410
	95% Confidence Interval for	Lower Bound	Statistic	15,47

Descriptives

				Status (0=NC,1=ADI) LC
	Mean	Upper Bound	Statistic	18,43
	5% Trimmed Mean		Statistic	17,28
	Median		Statistic	18,00
	Variance		Statistic	3,477
	Std. Deviation		Statistic	1,865
	Minimum		Statistic	14
	Maximum		Statistic	20
	Range		Statistic	6
	Interquartile Range		Statistic	4
	Skewness		Statistic	-,435
			Std. Error	,637
	Kurtosis		Statistic	-,873
			Std. Error	1,232
BIS	Mean		Statistic	18,5833
			Std. Error	,77321
	95% Confidence Interval for Mean	Lower Bound	Statistic	16,8815
		Upper Bound	Statistic	20,2852
	5% Trimmed Mean		Statistic	18,7593
	Median		Statistic	19,0000
	Variance		Statistic	7,174
	Std. Deviation		Statistic	2,67848
	Minimum		Statistic	12,00
	Maximum		Statistic	22,00
	Range		Statistic	10,00
	Interquartile Range		Statistic	3,75
	Skewness		Statistic	-1,269
			Std. Error	,637
	Kurtosis		Statistic	2,426
			Std. Error	1,232
ACTH1500	Mean		Statistic	,8575
			Std. Error	,02019
	95% Confidence Interval for	Lower Bound	Statistic	,8131

Descriptives

				Status (0=NC,1=ADI)
				OB
	Mean	Upper Bound	Statistic	17,15
	5% Trimmed Mean		Statistic	16,40
	Median		Statistic	16,50
	Variance		Statistic	4,716
	Std. Deviation		Statistic	2,172
	Minimum		Statistic	11
	Maximum		Statistic	20
	Range		Statistic	9
	Interquartile Range		Statistic	3
	Skewness		Statistic	-,657
			Std. Error	,441
	Kurtosis		Statistic	,227
			Std. Error	,858
BIS	Mean		Statistic	19,0357
			Std. Error	,71173
	95% Confidence Interval for Mean	Lower Bound	Statistic	17,5754
		Upper Bound	Statistic	20,4961
	5% Trimmed Mean		Statistic	19,1587
	Median		Statistic	19,0000
	Variance		Statistic	14,184
	Std. Deviation		Statistic	3,76615
	Minimum		Statistic	9,00
	Maximum		Statistic	26,00
	Range		Statistic	17,00
	Interquartile Range		Statistic	5,00
	Skewness		Statistic	-,317
			Std. Error	,441
	Kurtosis		Statistic	,468
			Std. Error	,858
ACTH1500	Mean		Statistic	,8982
			Std. Error	,03096
	95% Confidence Interval for	Lower Bound	Statistic	,8347

Descriptives

				Status (0=NC,1=ADI) LC
	Mean	Upper Bound	Statistic	,9019
	5% Trimmed Mean		Statistic	,8478
	Median		Statistic	,8300
	Variance		Statistic	,005
	Std. Deviation		Statistic	,06995
	Minimum		Statistic	,83
	Maximum		Statistic	1,06
	Range		Statistic	,23
	Interquartile Range		Statistic	,00
	Skewness		Statistic	2,702
			Std. Error	,637
	Kurtosis		Statistic	7,253
			Std. Error	1,232
ACTH1530_postCRH	Mean		Statistic	1,4800
			Std. Error	,15912
	95% Confidence Interval for Mean	Lower Bound	Statistic	1,1298
		Upper Bound	Statistic	1,8302
	5% Trimmed Mean		Statistic	1,4533
	Median		Statistic	1,4750
	Variance		Statistic	,304
	Std. Deviation		Statistic	,55120
	Minimum		Statistic	,83
	Maximum		Statistic	2,61
	Range		Statistic	1,78
	Interquartile Range		Statistic	,91
	Skewness		Statistic	,549
			Std. Error	,637
	Kurtosis		Statistic	-,069
			Std. Error	1,232
ACTH1545	Mean		Statistic	1,6175
			Std. Error	,24534
	95% Confidence Interval for	Lower Bound	Statistic	1,0775

Descriptives

				Status (0=NC,1=ADI)
				OB
	Mean	Upper Bound	Statistic	,9617
	5% Trimmed Mean		Statistic	,8702
	Median		Statistic	,8300
	Variance		Statistic	,027
	Std. Deviation		Statistic	,16382
	Minimum		Statistic	,83
	Maximum		Statistic	1,58
	Range		Statistic	,75
	Interquartile Range		Statistic	,04
	Skewness		Statistic	3,198
			Std. Error	,441
	Kurtosis		Statistic	11,277
			Std. Error	,858
ACTH1530_postCRH	Mean		Statistic	2,1182
			Std. Error	,30336
	95% Confidence Interval for Mean	Lower Bound	Statistic	1,4958
		Upper Bound	Statistic	2,7407
	5% Trimmed Mean		Statistic	1,8876
	Median		Statistic	1,6600
	Variance		Statistic	2,577
	Std. Deviation		Statistic	1,60524
	Minimum		Statistic	,83
	Maximum		Statistic	8,92
	Range		Statistic	8,09
	Interquartile Range		Statistic	1,21
	Skewness		Statistic	3,073
			Std. Error	,441
	Kurtosis		Statistic	11,857
			Std. Error	,858
ACTH1545	Mean		Statistic	2,0571
			Std. Error	,17266
	95% Confidence Interval for	Lower Bound	Statistic	1,7029

Descriptives

				Status (0=NC,1=ADI)
				LC
	Mean	Upper Bound	Statistic	2,1575
	5% Trimmed Mean		Statistic	1,5800
	Median		Statistic	1,4400
	Variance		Statistic	,722
	Std. Deviation		Statistic	,84989
	Minimum		Statistic	,83
	Maximum		Statistic	3,08
	Range		Statistic	2,25
	Interquartile Range		Statistic	1,53
	Skewness		Statistic	,883
			Std. Error	,637
	Kurtosis		Statistic	-,653
			Std. Error	1,232
ACTH1600	Mean		Statistic	2,0042
			Std. Error	,32171
	95% Confidence Interval for Mean	Lower Bound	Statistic	1,2961
		Upper Bound	Statistic	2,7123
	5% Trimmed Mean		Statistic	1,9419
	Median		Statistic	1,5700
	Variance		Statistic	1,242
	Std. Deviation		Statistic	1,11445
	Minimum		Statistic	,83
	Maximum		Statistic	4,30
	Range		Statistic	3,47
	Interquartile Range		Statistic	2,06
	Skewness		Statistic	,827
			Std. Error	,637
	Kurtosis		Statistic	-,216
			Std. Error	1,232
ACTH1615	Mean		Statistic	1,9608
			Std. Error	,32237
	95% Confidence Interval for	Lower Bound	Statistic	1,2513

Descriptives

				Status (0=NC,1=ADI)
				OB
	Mean	Upper Bound	Statistic	2,4114
	5% Trimmed Mean		Statistic	1,9930
	Median		Statistic	2,0250
	Variance		Statistic	,835
	Std. Deviation		Statistic	,91361
	Minimum		Statistic	,86
	Maximum		Statistic	4,56
	Range		Statistic	3,70
	Interquartile Range		Statistic	1,24
	Skewness		Statistic	,935
			Std. Error	,441
	Kurtosis		Statistic	,894
			Std. Error	,858
ACTH1600	Mean		Statistic	1,8239
			Std. Error	,12979
	95% Confidence Interval for Mean	Lower Bound	Statistic	1,5576
		Upper Bound	Statistic	2,0902
	5% Trimmed Mean		Statistic	1,7642
	Median		Statistic	1,7750
	Variance		Statistic	,472
	Std. Deviation		Statistic	,68678
	Minimum		Statistic	,97
	Maximum		Statistic	4,01
	Range		Statistic	3,04
	Interquartile Range		Statistic	,90
	Skewness		Statistic	1,315
			Std. Error	,441
	Kurtosis		Statistic	2,503
			Std. Error	,858
ACTH1615	Mean		Statistic	1,6486
			Std. Error	,11144
	95% Confidence Interval for	Lower Bound	Statistic	1,4199

Descriptives

				Status (0=NC,1=ADI)
				LC
	Mean	Upper Bound	Statistic	2,6704
	5% Trimmed Mean		Statistic	1,8820
	Median		Statistic	1,7200
	Variance		Statistic	1,247
	Std. Deviation		Statistic	1,11674
	Minimum		Statistic	,83
	Maximum		Statistic	4,51
	Range		Statistic	3,68
	Interquartile Range		Statistic	1,35
	Skewness		Statistic	1,289
			Std. Error	,637
	Kurtosis		Statistic	1,385
			Std. Error	1,232
CRT1500	Mean		Statistic	21,3583
			Std. Error	5,08450
	95% Confidence Interval for Mean	Lower Bound	Statistic	10,1674
		Upper Bound	Statistic	32,5493
	5% Trimmed Mean		Statistic	19,5037
	Median		Statistic	15,8000
	Variance		Statistic	310,226
	Std. Deviation		Statistic	17,61324
	Minimum		Statistic	6,90
	Maximum		Statistic	69,20
	Range		Statistic	62,30
	Interquartile Range		Statistic	13,28
	Skewness		Statistic	2,146
			Std. Error	,637
	Kurtosis		Statistic	4,927
			Std. Error	1,232
CRT1530_postCRH	Mean		Statistic	29,5333
			Std. Error	4,28757
	95% Confidence Interval for	Lower Bound	Statistic	20,0965

Descriptives

				Status (0=NC,1=ADI)
				OB
	Mean	Upper Bound	Statistic	1,8772
	5% Trimmed Mean		Statistic	1,6173
	Median		Statistic	1,7250
	Variance		Statistic	,348
	Std. Deviation		Statistic	,58967
	Minimum		Statistic	,83
	Maximum		Statistic	3,19
	Range		Statistic	2,36
	Interquartile Range		Statistic	,90
	Skewness		Statistic	,448
			Std. Error	,441
	Kurtosis		Statistic	,175
			Std. Error	,858
CRT1500	Mean		Statistic	20,7571
			Std. Error	1,60991
	95% Confidence Interval for Mean	Lower Bound	Statistic	17,4539
		Upper Bound	Statistic	24,0604
	5% Trimmed Mean		Statistic	20,0849
	Median		Statistic	19,9500
	Variance		Statistic	72,571
	Std. Deviation		Statistic	8,51884
	Minimum		Statistic	5,90
	Maximum		Statistic	48,60
	Range		Statistic	42,70
	Interquartile Range		Statistic	8,10
	Skewness		Statistic	1,515
			Std. Error	,441
	Kurtosis		Statistic	3,819
			Std. Error	,858
CRT1530_postCRH	Mean		Statistic	98,8393
			Std. Error	19,06088
	95% Confidence Interval for	Lower Bound	Statistic	59,7296

Descriptives

				Status (0=NC,1=ADI) LC
	Mean	Upper Bound	Statistic	38,9702
	5% Trimmed Mean		Statistic	28,5204
	Median		Statistic	25,5000
	Variance		Statistic	220,599
	Std. Deviation		Statistic	14,85257
	Minimum		Statistic	11,80
	Maximum		Statistic	65,50
	Range		Statistic	53,70
	Interquartile Range		Statistic	18,00
	Skewness		Statistic	1,303
			Std. Error	,637
	Kurtosis		Statistic	2,063
			Std. Error	1,232
CRT1545	Mean		Statistic	43,0500
			Std. Error	7,25367
	95% Confidence Interval for Mean	Lower Bound	Statistic	27,0848
		Upper Bound	Statistic	59,0152
	5% Trimmed Mean		Statistic	42,4278
	Median		Statistic	39,2000
	Variance		Statistic	631,388
	Std. Deviation		Statistic	25,12744
	Minimum		Statistic	13,10
	Maximum		Statistic	84,20
	Range		Statistic	71,10
	Interquartile Range		Statistic	41,43
	Skewness		Statistic	,455
			Std. Error	,637
	Kurtosis		Statistic	-1,215
			Std. Error	1,232
CRT1600	Mean		Statistic	54,5750
			Std. Error	9,72706
	95% Confidence Interval for	Lower Bound	Statistic	33,1659

Descriptives

				Status (0=NC,1=ADI)
				OB
	Mean	Upper Bound	Statistic	137,9490
	5% Trimmed Mean		Statistic	89,2127
	Median		Statistic	48,9000
	Variance		Statistic	10172,882
	Std. Deviation		Statistic	100,86071
	Minimum		Statistic	10,20
	Maximum		Statistic	364,70
	Range		Statistic	354,50
	Interquartile Range		Statistic	121,58
	Skewness		Statistic	1,454
			Std. Error	,441
	Kurtosis		Statistic	1,310
			Std. Error	,858
CRT1545	Mean		Statistic	105,6000
			Std. Error	20,05385
	95% Confidence Interval for Mean	Lower Bound	Statistic	64,4529
		Upper Bound	Statistic	146,7471
	5% Trimmed Mean		Statistic	97,5373
	Median		Statistic	56,7000
	Variance		Statistic	11260,396
	Std. Deviation		Statistic	106,11501
	Minimum		Statistic	10,90
	Maximum		Statistic	350,80
	Range		Statistic	339,90
	Interquartile Range		Statistic	120,00
	Skewness		Statistic	1,220
			Std. Error	,441
	Kurtosis		Statistic	,255
			Std. Error	,858
CRT1600	Mean		Statistic	110,0786
			Std. Error	19,91382
	95% Confidence Interval for	Lower Bound	Statistic	69,2188

Descriptives

				Status (0=NC,1=ADI)
				LC
	Mean	Upper Bound	Statistic	75,9841
	5% Trimmed Mean		Statistic	53,3611
	Median		Statistic	50,7000
	Variance		Statistic	1135,388
	Std. Deviation		Statistic	33,69551
	Minimum		Statistic	19,40
	Maximum		Statistic	111,60
	Range		Statistic	92,20
	Interquartile Range		Statistic	59,45
	Skewness		Statistic	,509
			Std. Error	,637
	Kurtosis		Statistic	-1,209
			Std. Error	1,232
CRT1615	Mean		Statistic	66,7167
			Std. Error	12,23283
	95% Confidence Interval for Mean	Lower Bound	Statistic	39,7924
		Upper Bound	Statistic	93,6410
	5% Trimmed Mean		Statistic	64,6296
	Median		Statistic	66,3500
	Variance		Statistic	1795,707
	Std. Deviation		Statistic	42,37578
	Minimum		Statistic	18,90
	Maximum		Statistic	152,10
	Range		Statistic	133,20
	Interquartile Range		Statistic	75,75
	Skewness		Statistic	,646
			Std. Error	,637
	Kurtosis		Statistic	-,349
			Std. Error	1,232
Stress-ACTH-max	Mean		Statistic	2,1708
			Std. Error	,32389
	95% Confidence Interval for	Lower Bound	Statistic	1,4580

Descriptives

				Status (0=NC,1=ADI)
				OB
	Mean	Upper Bound	Statistic	150,9384
	5% Trimmed Mean		Statistic	100,7444
	Median		Statistic	64,3500
	Variance		Statistic	11103,685
	Std. Deviation		Statistic	105,37402
	Minimum		Statistic	12,40
	Maximum		Statistic	410,40
	Range		Statistic	398,00
	Interquartile Range		Statistic	142,40
	Skewness		Statistic	1,254
			Std. Error	,441
	Kurtosis		Statistic	,914
			Std. Error	,858
CRT1615	Mean		Statistic	95,8571
			Std. Error	17,55462
	95% Confidence Interval for Mean	Lower Bound	Statistic	59,8380
		Upper Bound	Statistic	131,8763
	5% Trimmed Mean		Statistic	87,0452
	Median		Statistic	59,6500
	Variance		Statistic	8628,613
	Std. Deviation		Statistic	92,89033
	Minimum		Statistic	13,70
	Maximum		Statistic	360,00
	Range		Statistic	346,30
	Interquartile Range		Statistic	120,10
	Skewness		Statistic	1,394
			Std. Error	,441
	Kurtosis		Statistic	1,239
			Std. Error	,858
Stress-ACTH-max	Mean		Statistic	2,5079
			Std. Error	,29541
	95% Confidence Interval for	Lower Bound	Statistic	1,9017

Descriptives

				Status (0=NC,1=ADI)
				LC
	Mean	Upper Bound	Statistic	2,8837
	5% Trimmed Mean		Statistic	2,1126
	Median		Statistic	1,8500
	Variance		Statistic	1,259
	Std. Deviation		Statistic	1,12199
	Minimum		Statistic	,88
	Maximum		Statistic	4,51
	Range		Statistic	3,63
	Interquartile Range		Statistic	1,72
	Skewness		Statistic	,875
			Std. Error	,637
	Kurtosis		Statistic	,036
			Std. Error	1,232
Stress-ACTH-delta-max	Mean		Statistic	1,3133
			Std. Error	,32429
	95% Confidence Interval for Mean	Lower Bound	Statistic	,5996
		Upper Bound	Statistic	2,0271
	5% Trimmed Mean		Statistic	1,2520
	Median		Statistic	1,0200
	Variance		Statistic	1,262
	Std. Deviation		Statistic	1,12337
	Minimum		Statistic	,05
	Maximum		Statistic	3,68
	Range		Statistic	3,63
	Interquartile Range		Statistic	1,76
	Skewness		Statistic	,894
			Std. Error	,637
	Kurtosis		Statistic	,070
			Std. Error	1,232
Stress-A-AUC	Mean		Statistic	6,51083
			Std. Error	,839522
	95% Confidence Interval for	Lower Bound	Statistic	4,66306

Descriptives

				Status (0=NC,1=ADI)
				OB
	Mean	Upper Bound	Statistic	3,1140
	5% Trimmed Mean		Statistic	2,3036
	Median		Statistic	2,1050
	Variance		Statistic	2,443
	Std. Deviation		Statistic	1,56314
	Minimum		Statistic	,97
	Maximum		Statistic	8,92
	Range		Statistic	7,95
	Interquartile Range		Statistic	1,40
	Skewness		Statistic	2,767
			Std. Error	,441
	Kurtosis		Statistic	10,124
			Std. Error	,858
Stress-ACTH-delta-max	Mean		Statistic	1,6096
			Std. Error	,30291
	95% Confidence Interval for Mean	Lower Bound	Statistic	,9881
		Upper Bound	Statistic	2,2312
	5% Trimmed Mean		Statistic	1,4235
	Median		Statistic	1,2250
	Variance		Statistic	2,569
	Std. Deviation		Statistic	1,60283
	Minimum		Statistic	-,44
	Maximum		Statistic	8,09
	Range		Statistic	8,53
	Interquartile Range		Statistic	1,41
	Skewness		Statistic	2,616
			Std. Error	,441
	Kurtosis		Statistic	9,469
			Std. Error	,858
Stress-A-AUC	Mean		Statistic	7,27339
			Std. Error	,517565
	95% Confidence Interval for	Lower Bound	Statistic	6,21144

Descriptives

				Status (0=NC,1=ADI) LC
	Mean	Upper Bound	Statistic	8,35861
	5% Trimmed Mean		Statistic	6,38509
	Median		Statistic	5,52750
	Variance		Statistic	8,458
	Std. Deviation		Statistic	2,908188
	Minimum		Statistic	3,365
	Maximum		Statistic	11,920
	Range		Statistic	8,555
	Interquartile Range		Statistic	4,765
	Skewness		Statistic	,801
			Std. Error	,637
	Kurtosis		Statistic	-,523
			Std. Error	1,232
Stress-C-max	Mean		Statistic	67,2333
			Std. Error	12,06751
	95% Confidence Interval for Mean	Lower Bound	Statistic	40,6729
		Upper Bound	Statistic	93,7937
	5% Trimmed Mean		Statistic	65,0648
	Median		Statistic	66,3500
	Variance		Statistic	1747,497
	Std. Deviation		Statistic	41,80307
	Minimum		Statistic	21,40
	Maximum		Statistic	152,10
	Range		Statistic	130,70
	Interquartile Range		Statistic	74,83
	Skewness		Statistic	,679
			Std. Error	,637
	Kurtosis		Statistic	-,293
			Std. Error	1,232
Stress-C-dmax	Mean		Statistic	45,8750
			Std. Error	14,06442
	95% Confidence Interval for	Lower Bound	Statistic	14,9194

Descriptives

				Status (0=NC,1=ADI)
				OB
	Mean	Upper Bound	Statistic	8,33535
	5% Trimmed Mean		Statistic	7,11758
	Median		Statistic	6,77500
	Variance		Statistic	7,500
	Std. Deviation		Statistic	2,738694
	Minimum		Statistic	3,490
	Maximum		Statistic	14,360
	Range		Statistic	10,870
	Interquartile Range		Statistic	4,174
	Skewness		Statistic	,803
			Std. Error	,441
	Kurtosis		Statistic	,195
			Std. Error	,858
Stress-C-max	Mean		Statistic	121,9536
			Std. Error	21,49043
	95% Confidence Interval for Mean	Lower Bound	Statistic	77,8588
		Upper Bound	Statistic	166,0483
	5% Trimmed Mean		Statistic	112,6016
	Median		Statistic	74,3500
	Variance		Statistic	12931,481
	Std. Deviation		Statistic	113,71667
	Minimum		Statistic	14,10
	Maximum		Statistic	410,40
	Range		Statistic	396,30
	Interquartile Range		Statistic	131,97
	Skewness		Statistic	1,191
			Std. Error	,441
	Kurtosis		Statistic	,472
			Std. Error	,858
Stress-C-dmax	Mean		Statistic	101,1964
			Std. Error	20,91027
	95% Confidence Interval for	Lower Bound	Statistic	58,2921

Descriptives

				Status (0=NC,1=ADI)
				LC
	Mean	Upper Bound	Statistic	76,8306
	5% Trimmed Mean		Statistic	43,1222
	Median		Statistic	25,4500
	Variance		Statistic	2373,697
	Std. Deviation		Statistic	48,72060
	Minimum		Statistic	-3,90
	Maximum		Statistic	145,20
	Range		Statistic	149,10
	Interquartile Range		Statistic	82,28
	Skewness		Statistic	,867
			Std. Error	,637
	Kurtosis		Statistic	-,357
			Std. Error	1,232
Stress-C-AUC	Mean		Statistic	171,1958
			Std. Error	25,33125
	95% Confidence Interval for Mean	Lower Bound	Statistic	115,4421
		Upper Bound	Statistic	226,9495
	5% Trimmed Mean		Statistic	170,8509
	Median		Statistic	165,3750
	Variance		Statistic	7700,066
	Std. Deviation		Statistic	87,75002
	Minimum		Statistic	62,75
	Maximum		Statistic	285,85
	Range		Statistic	223,10
	Interquartile Range		Statistic	174,68
	Skewness		Statistic	,093
			Std. Error	,637
	Kurtosis		Statistic	-1,997
			Std. Error	1,232
AC_ratio_postCRH_1530	Mean		Statistic	,06126217
			Std. Error	,009609645
	95% Confidence Interval for	Lower Bound	Statistic	,04011149

Descriptives

				Status (0=NC,1=ADI)
				OB
	Mean	Upper Bound	Statistic	144,1008
	5% Trimmed Mean		Statistic	92,2944
	Median		Statistic	44,2000
	Variance		Statistic	12242,704
	Std. Deviation		Statistic	110,64675
	Minimum		Statistic	-,70
	Maximum		Statistic	370,50
	Range		Statistic	371,20
	Interquartile Range		Statistic	124,80
	Skewness		Statistic	1,160
			Std. Error	,441
	Kurtosis		Statistic	,263
			Std. Error	,858
Stress-C-AUC	Mean		Statistic	372,8250
			Std. Error	66,45269
	95% Confidence Interval for Mean	Lower Bound	Statistic	236,4753
		Upper Bound	Statistic	509,1747
	5% Trimmed Mean		Statistic	343,0996
	Median		Statistic	216,0000
	Variance		Statistic	123646,873
	Std. Deviation		Statistic	351,63457
	Minimum		Statistic	48,60
	Maximum		Statistic	1308,35
	Range		Statistic	1259,75
	Interquartile Range		Statistic	411,30
	Skewness		Statistic	1,249
			Std. Error	,441
	Kurtosis		Statistic	,621
			Std. Error	,858
AC_ratio_postCRH_1530	Mean		Statistic	,05621411
			Std. Error	,022277085
	95% Confidence Interval for	Lower Bound	Statistic	,01050530

Descriptives

				Status (0=NC,1=ADI)
				LC
	Mean	Upper Bound	Statistic	,08241286
	5% Trimmed Mean		Statistic	,06086135
	Median		Statistic	,05893849
	Variance		Statistic	,001
	Std. Deviation		Statistic	,033288788
	Minimum		Statistic	,019213
	Maximum		Statistic	,110526
	Range		Statistic	,091313
	Interquartile Range		Statistic	,063121
	Skewness		Statistic	,302
			Std. Error	,637
	Kurtosis		Statistic	-1,350
			Std. Error	1,232
AC_ratio_MAX	Mean		Statistic	,039807
			Std. Error	,0058109
	95% Confidence Interval for Mean	Lower Bound	Statistic	,027017
		Upper Bound	Statistic	,052596
	5% Trimmed Mean		Statistic	,039600
	Median		Statistic	,037810
	Variance		Statistic	,000
	Std. Deviation		Statistic	,0201296
	Minimum		Statistic	,0132
	Maximum		Statistic	,0701
	Range		Statistic	,0569
	Interquartile Range		Statistic	,0408
	Skewness		Statistic	,339
			Std. Error	,637
	Kurtosis		Statistic	-1,188
			Std. Error	1,232
AC_ratio_AUC	Mean		Statistic	,045963
			Std. Error	,0067647
	95% Confidence Interval for	Lower Bound	Statistic	,031074

Descriptives

				Status (0=NC,1=ADI)
				OB
	Mean	Upper Bound	Statistic	,10192291
	5% Trimmed Mean		Statistic	,03468203
	Median		Statistic	,02850999
	Variance		Statistic	,014
	Std. Deviation		Statistic	,117879256
	Minimum		Statistic	,007526
	Maximum		Statistic	,641727
	Range		Statistic	,634201
	Interquartile Range		Statistic	,037008
	Skewness		Statistic	4,868
			Std. Error	,441
	Kurtosis		Statistic	24,813
			Std. Error	,858
AC_ratio_MAX	Mean		Statistic	,055489
			Std. Error	,0220713
	95% Confidence Interval for Mean	Lower Bound	Statistic	,010202
		Upper Bound	Statistic	,100775
	5% Trimmed Mean		Statistic	,034151
	Median		Statistic	,023225
	Variance		Statistic	,014
	Std. Deviation		Statistic	,1167906
	Minimum		Statistic	,0072
	Maximum		Statistic	,6326
	Range		Statistic	,6255
	Interquartile Range		Statistic	,0340
	Skewness		Statistic	4,798
			Std. Error	,441
	Kurtosis		Statistic	24,244
			Std. Error	,858
AC_ratio_AUC	Mean		Statistic	,041641
			Std. Error	,0083667
	95% Confidence Interval for	Lower Bound	Statistic	,024474

Descriptives

				Status (0=NC,1=ADI) LC
	Mean	Upper Bound	Statistic	,060852
	5% Trimmed Mean		Statistic	,045203
	Median		Statistic	,042860
	Variance		Statistic	,001
	Std. Deviation		Statistic	,0234335
	Minimum		Statistic	,0149
	Maximum		Statistic	,0907
	Range		Statistic	,0758
	Interquartile Range		Statistic	,0388
	Skewness		Statistic	,452
			Std. Error	,637
	Kurtosis		Statistic	-,485
			Std. Error	1,232
VOI-BS_NUK_Group	Mean		Statistic	,5556078
			Std. Error	,04493613
	95% Confidence Interval for Mean	Lower Bound	Statistic	,4567041
		Upper Bound	Statistic	,6545116
	5% Trimmed Mean		Statistic	,5667500
	Median		Statistic	,5612280
	Variance		Statistic	,024
	Std. Deviation		Statistic	,15566333
	Minimum		Statistic	,17588
	Maximum		Statistic	,73478
	Range		Statistic	,55891
	Interquartile Range		Statistic	,22545
	Skewness		Statistic	-1,299
			Std. Error	,637
	Kurtosis		Statistic	2,221
			Std. Error	1,232
Frontal cortex	Mean		Statistic	,2153972
			Std. Error	,02094943
	95% Confidence Interval for	Lower Bound	Statistic	,1692878

Descriptives

				Status (0=NC,1=ADI) OB
	Mean	Upper Bound	Statistic	,058808
	5% Trimmed Mean		Statistic	,035422
	Median		Statistic	,025295
	Variance		Statistic	,002
	Std. Deviation		Statistic	,0442723
	Minimum		Statistic	,0073
	Maximum		Statistic	,2172
	Range		Statistic	,2099
	Interquartile Range		Statistic	,0387
	Skewness		Statistic	2,652
			Std. Error	,441
	Kurtosis		Statistic	8,705
			Std. Error	,858
VOI-BS_NUK_Group	Mean		Statistic	,6109611
			Std. Error	,03106560
	95% Confidence Interval for Mean	Lower Bound	Statistic	,5472198
		Upper Bound	Statistic	,6747024
	5% Trimmed Mean		Statistic	,6168290
	Median		Statistic	,6356855
	Variance		Statistic	,027
	Std. Deviation		Statistic	,16438369
	Minimum		Statistic	,26265
	Maximum		Statistic	,84110
	Range		Statistic	,57845
	Interquartile Range		Statistic	,26216
	Skewness		Statistic	-,566
			Std. Error	,441
	Kurtosis		Statistic	-,487
			Std. Error	,858
Frontal cortex	Mean		Statistic	,2191100
			Std. Error	,02002024
	95% Confidence Interval for	Lower Bound	Statistic	,1780318

Descriptives

				Status (0=NC,1=ADI)
				LC
	Mean	Upper Bound	Statistic	,2615066
	5% Trimmed Mean		Statistic	,2202381
	Median		Statistic	,2291548
	Variance		Statistic	,005
	Std. Deviation		Statistic	,07257095
	Minimum		Statistic	,03198
	Maximum		Statistic	,31168
	Range		Statistic	,27970
	Interquartile Range		Statistic	,08617
	Skewness		Statistic	-1,447
			Std. Error	,637
	Kurtosis		Statistic	3,100
			Std. Error	1,232
Orbitofronta cortex	Mean		Statistic	,4140943
			Std. Error	,03242915
	95% Confidence Interval for Mean	Lower Bound	Statistic	,3427182
		Upper Bound	Statistic	,4854704
	5% Trimmed Mean		Statistic	,4209205
	Median		Statistic	,4197808
	Variance		Statistic	,013
	Std. Deviation		Statistic	,11233788
	Minimum		Statistic	,15268
	Maximum		Statistic	,55264
	Range		Statistic	,39997
	Interquartile Range		Statistic	,15704
	Skewness		Statistic	-1,082
			Std. Error	,637
	Kurtosis		Statistic	1,380
			Std. Error	1,232
Dorsolateral prefrontal cortex	Mean		Statistic	,2241005
			Std. Error	,02100611
	95% Confidence Interval for	Lower Bound	Statistic	,1778663

Descriptives

				Status (0=NC,1=ADI) OB
	Mean	Upper Bound	Statistic	,2601881
	5% Trimmed Mean		Statistic	,2207187
	Median		Statistic	,2252228
	Variance		Statistic	,011
	Std. Deviation		Statistic	,10593718
	Minimum		Statistic	-,00571
	Maximum		Statistic	,40843
	Range		Statistic	,41414
	Interquartile Range		Statistic	,10167
	Skewness		Statistic	-,095
			Std. Error	,441
	Kurtosis		Statistic	-,157
			Std. Error	,858
Orbitofronta cortex	Mean		Statistic	,4532688
			Std. Error	,02789668
	95% Confidence Interval for Mean	Lower Bound	Statistic	,3960295
		Upper Bound	Statistic	,5105081
	5% Trimmed Mean		Statistic	,4538937
	Median		Statistic	,4366905
	Variance		Statistic	,022
	Std. Deviation		Statistic	,14761535
	Minimum		Statistic	,19726
	Maximum		Statistic	,69857
	Range		Statistic	,50132
	Interquartile Range		Statistic	,24423
	Skewness		Statistic	-,007
			Std. Error	,441
	Kurtosis		Statistic	-,936
			Std. Error	,858
Dorsolateral prefrontal cortex	Mean		Statistic	,2389408
			Std. Error	,02085973
	95% Confidence Interval for	Lower Bound	Statistic	,1961402

Descriptives

				Status (0=NC,1=ADI)
				LC
	Mean	Upper Bound	Statistic	,2703346
	5% Trimmed Mean		Statistic	,2280446
	Median		Statistic	,2258988
	Variance		Statistic	,005
	Std. Deviation		Statistic	,07276732
	Minimum		Statistic	,06130
	Maximum		Statistic	,31590
	Range		Statistic	,25460
	Interquartile Range		Statistic	,09654
	Skewness		Statistic	-,887
			Std. Error	,637
	Kurtosis		Statistic	1,096
			Std. Error	1,232
Anterior cingulate cortex	Mean		Statistic	,3905384
			Std. Error	,03168859
	95% Confidence Interval for Mean	Lower Bound	Statistic	,3207923
		Upper Bound	Statistic	,4602845
	5% Trimmed Mean		Statistic	,4002293
	Median		Statistic	,3990678
	Variance		Statistic	,012
	Std. Deviation		Statistic	,10977249
	Minimum		Statistic	,08280
	Maximum		Statistic	,52384
	Range		Statistic	,44104
	Interquartile Range		Statistic	,09300
	Skewness		Statistic	-2,104
			Std. Error	,637
	Kurtosis		Statistic	6,165
			Std. Error	1,232
Insula	Mean		Statistic	,6484520
			Std. Error	,04316724
	95% Confidence Interval for	Lower Bound	Statistic	,5534415

Descriptives

				Status (0=NC,1=ADI)
				OB
	Mean	Upper Bound	Statistic	,2817414
	5% Trimmed Mean		Statistic	,2380577
	Median		Statistic	,2310983
	Variance		Statistic	,012
	Std. Deviation		Statistic	,11037932
	Minimum		Statistic	,05043
	Maximum		Statistic	,44251
	Range		Statistic	,39208
	Interquartile Range		Statistic	,17001
	Skewness		Statistic	,176
			Std. Error	,441
	Kurtosis		Statistic	-,731
			Std. Error	,858
Anterior cingulate cortex	Mean		Statistic	,4061846
			Std. Error	,02303176
	95% Confidence Interval for Mean	Lower Bound	Statistic	,3589273
		Upper Bound	Statistic	,4534419
	5% Trimmed Mean		Statistic	,4080584
	Median		Statistic	,4044560
	Variance		Statistic	,015
	Std. Deviation		Statistic	,12187264
	Minimum		Statistic	,16771
	Maximum		Statistic	,61102
	Range		Statistic	,44331
	Interquartile Range		Statistic	,19647
	Skewness		Statistic	-,097
			Std. Error	,441
	Kurtosis		Statistic	-,648
			Std. Error	,858
Insula	Mean		Statistic	,6552392
			Std. Error	,03278435
	95% Confidence Interval for	Lower Bound	Statistic	,5879712

Descriptives

				Status (0=NC,1=ADI)
				LC
	Mean	Upper Bound	Statistic	,7434625
	5% Trimmed Mean		Statistic	,6584200
	Median		Statistic	,6997405
	Variance		Statistic	,022
	Std. Deviation		Statistic	,14953572
	Minimum		Statistic	,29008
	Maximum		Statistic	,82740
	Range		Statistic	,53732
	Interquartile Range		Statistic	,17509
	Skewness		Statistic	-1,419
			Std. Error	,637
	Kurtosis		Statistic	1,990
			Std. Error	1,232
Hippocampus	Mean		Statistic	,5469163
			Std. Error	,04268178
	95% Confidence Interval for Mean	Lower Bound	Statistic	,4529744
		Upper Bound	Statistic	,6408583
	5% Trimmed Mean		Statistic	,5576546
	Median		Statistic	,5535085
	Variance		Statistic	,022
	Std. Deviation		Statistic	,14785403
	Minimum		Statistic	,17784
	Maximum		Statistic	,72271
	Range		Statistic	,54487
	Interquartile Range		Statistic	,17388
	Skewness		Statistic	-1,381
			Std. Error	,637
	Kurtosis		Statistic	2,826
			Std. Error	1,232
Amygdala	Mean		Statistic	1,2559004
			Std. Error	,10270062
	95% Confidence Interval for	Lower Bound	Statistic	1,0298578

Descriptives

				Status (0=NC,1=ADI)
				OB
	Mean	Upper Bound	Statistic	,7225071
	5% Trimmed Mean		Statistic	,6604555
	Median		Statistic	,6621830
	Variance		Statistic	,030
	Std. Deviation		Statistic	,17347846
	Minimum		Statistic	,28458
	Maximum		Statistic	,93205
	Range		Statistic	,64747
	Interquartile Range		Statistic	,25440
	Skewness		Statistic	-,345
			Std. Error	,441
	Kurtosis		Statistic	-,349
			Std. Error	,858
Hippocampus	Mean		Statistic	,5816388
			Std. Error	,05076563
	95% Confidence Interval for Mean	Lower Bound	Statistic	,4774764
		Upper Bound	Statistic	,6858013
	5% Trimmed Mean		Statistic	,5657312
	Median		Statistic	,5663113
	Variance		Statistic	,072
	Std. Deviation		Statistic	,26862649
	Minimum		Statistic	,02843
	Maximum		Statistic	1,57130
	Range		Statistic	1,54287
	Interquartile Range		Statistic	,24722
	Skewness		Statistic	1,543
			Std. Error	,441
	Kurtosis		Statistic	6,442
			Std. Error	,858
Amygdala	Mean		Statistic	1,3239375
			Std. Error	,07361257
	95% Confidence Interval for	Lower Bound	Statistic	1,1728970

Descriptives

				Status (0=NC,1=ADI)
				LC
	Mean	Upper Bound	Statistic	1,4819429
	5% Trimmed Mean		Statistic	1,2856566
	Median		Statistic	1,3604425
	Variance		Statistic	,127
	Std. Deviation		Statistic	,35576538
	Minimum		Statistic	,28824
	Maximum		Statistic	1,68795
	Range		Statistic	1,39971
	Interquartile Range		Statistic	,35354
	Skewness		Statistic	-1,960
			Std. Error	,637
	Kurtosis		Statistic	5,028
			Std. Error	1,232
Ncl. accumbens	Mean		Statistic	1,6306270
			Std. Error	,10884520
	95% Confidence Interval for Mean	Lower Bound	Statistic	1,3910604
		Upper Bound	Statistic	1,8701937
	5% Trimmed Mean		Statistic	1,6728704
	Median		Statistic	1,7360913
	Variance		Statistic	,142
	Std. Deviation		Statistic	,37705082
	Minimum		Statistic	,54303
	Maximum		Statistic	1,95784
	Range		Statistic	1,41481
	Interquartile Range		Statistic	,24018
	Skewness		Statistic	-2,489
			Std. Error	,637
	Kurtosis		Statistic	7,084
			Std. Error	1,232
Head of the caudate	Mean		Statistic	1,4615196
			Std. Error	,10649500
	95% Confidence Interval for	Lower Bound	Statistic	1,2271257

Descriptives

				Status (0=NC,1=ADI) OB
	Mean	Upper Bound	Statistic	1,4749780
	5% Trimmed Mean		Statistic	1,3256755
	Median		Statistic	1,4271800
	Variance		Statistic	,152
	Std. Deviation		Statistic	,38952109
	Minimum		Statistic	,45642
	Maximum		Statistic	2,26939
	Range		Statistic	1,81296
	Interquartile Range		Statistic	,42468
	Skewness		Statistic	-,416
			Std. Error	,441
	Kurtosis		Statistic	1,277
			Std. Error	,858
Ncl. accumbens	Mean		Statistic	1,5524620
			Std. Error	,07925888
	95% Confidence Interval for Mean	Lower Bound	Statistic	1,3898362
		Upper Bound	Statistic	1,7150878
	5% Trimmed Mean		Statistic	1,5536204
	Median		Statistic	1,5502880
	Variance		Statistic	,176
	Std. Deviation		Statistic	,41939859
	Minimum		Statistic	,74888
	Maximum		Statistic	2,34830
	Range		Statistic	1,59942
	Interquartile Range		Statistic	,46777
	Skewness		Statistic	-,249
			Std. Error	,441
	Kurtosis		Statistic	-,192
			Std. Error	,858
Head of the caudate	Mean		Statistic	1,4537705
			Std. Error	,06759457
	95% Confidence Interval for	Lower Bound	Statistic	1,3150779

Descriptives

				Status (0=NC,1=ADI)
				LC
	Mean	Upper Bound	Statistic	1,6959135
	5% Trimmed Mean		Statistic	1,4804187
	Median		Statistic	1,4662500
	Variance		Statistic	,136
	Std. Deviation		Statistic	,36890950
	Minimum		Statistic	,65169
	Maximum		Statistic	1,93116
	Range		Statistic	1,27947
	Interquartile Range		Statistic	,48648
	Skewness		Statistic	-,861
			Std. Error	,637
	Kurtosis		Statistic	,632
			Std. Error	1,232
Putamen	Mean		Statistic	1,2951137
			Std. Error	,10117147
	95% Confidence Interval for Mean	Lower Bound	Statistic	1,0724368
		Upper Bound	Statistic	1,5177907
	5% Trimmed Mean		Statistic	1,3198973
	Median		Statistic	1,2606627
	Variance		Statistic	,123
	Std. Deviation		Statistic	,35046826
	Minimum		Statistic	,49676
	Maximum		Statistic	1,64737
	Range		Statistic	1,15061
	Interquartile Range		Statistic	,51581
	Skewness		Statistic	-,949
			Std. Error	,637
	Kurtosis		Statistic	,929
			Std. Error	1,232
Thalamus	Mean		Statistic	1,4488371
			Std. Error	,10986845
	95% Confidence Interval for	Lower Bound	Statistic	1,2070183

Descriptives

				Status (0=NC,1=ADI) OB
	Mean	Upper Bound	Statistic	1,5924631
	5% Trimmed Mean		Statistic	1,4663921
	Median		Statistic	1,4995915
	Variance		Statistic	,128
	Std. Deviation		Statistic	,35767684
	Minimum		Statistic	,65048
	Maximum		Statistic	2,02511
	Range		Statistic	1,37464
	Interquartile Range		Statistic	,44417
	Skewness		Statistic	-,602
			Std. Error	,441
	Kurtosis		Statistic	-,216
			Std. Error	,858
Putamen	Mean		Statistic	1,2522039
			Std. Error	,05441184
	95% Confidence Interval for Mean	Lower Bound	Statistic	1,1405600
		Upper Bound	Statistic	1,3638477
	5% Trimmed Mean		Statistic	1,2499927
	Median		Statistic	1,2534310
	Variance		Statistic	,083
	Std. Deviation		Statistic	,28792038
	Minimum		Statistic	,70724
	Maximum		Statistic	1,84789
	Range		Statistic	1,14064
	Interquartile Range		Statistic	,44019
	Skewness		Statistic	,037
			Std. Error	,441
	Kurtosis		Statistic	-,416
			Std. Error	,858
Thalamus	Mean		Statistic	1,4891469
			Std. Error	,07347556
	95% Confidence Interval for	Lower Bound	Statistic	1,3383875

Descriptives

				Status (0=NC,1=ADI)
				LC
	Mean	Upper Bound	Statistic	1,6906559
	5% Trimmed Mean		Statistic	1,4550347
	Median		Statistic	1,3569188
	Variance		Statistic	,145
	Std. Deviation		Statistic	,38059547
	Minimum		Statistic	,72601
	Maximum		Statistic	2,06011
	Range		Statistic	1,33410
	Interquartile Range		Statistic	,56835
	Skewness		Statistic	,065
			Std. Error	,637
	Kurtosis		Statistic	-,013
			Std. Error	1,232
Hypothalamus	Mean		Statistic	1,8662988
			Std. Error	,15744953
	95% Confidence Interval for Mean	Lower Bound	Statistic	1,5197547
		Upper Bound	Statistic	2,2128429
	5% Trimmed Mean		Statistic	1,9065256
	Median		Statistic	1,9523408
	Variance		Statistic	,297
	Std. Deviation		Statistic	,54542118
	Minimum		Statistic	,41374
	Maximum		Statistic	2,59477
	Range		Statistic	2,18103
	Interquartile Range		Statistic	,52041
	Skewness		Statistic	-1,784
			Std. Error	,637
	Kurtosis		Statistic	4,523
			Std. Error	1,232
Substantia nigra	Mean		Statistic	1,5324688
			Std. Error	,14214439
	95% Confidence Interval for	Lower Bound	Statistic	1,2196110

Descriptives

				Status (0=NC,1=ADI)
				OB
	Mean	Upper Bound	Statistic	1,6399063
	5% Trimmed Mean		Statistic	1,4779726
	Median		Statistic	1,4542458
	Variance		Statistic	,151
	Std. Deviation		Statistic	,38879613
	Minimum		Statistic	,76241
	Maximum		Statistic	2,45616
	Range		Statistic	1,69375
	Interquartile Range		Statistic	,43492
	Skewness		Statistic	,644
			Std. Error	,441
	Kurtosis		Statistic	,313
			Std. Error	,858
Hypothalamus	Mean		Statistic	2,0580667
			Std. Error	,10624734
	95% Confidence Interval for Mean	Lower Bound	Statistic	1,8400652
		Upper Bound	Statistic	2,2760683
	5% Trimmed Mean		Statistic	2,0883282
	Median		Statistic	2,1893582
	Variance		Statistic	,316
	Std. Deviation		Statistic	,56220809
	Minimum		Statistic	,65890
	Maximum		Statistic	2,96032
	Range		Statistic	2,30142
	Interquartile Range		Statistic	,64901
	Skewness		Statistic	-1,020
			Std. Error	,441
	Kurtosis		Statistic	1,140
			Std. Error	,858
Substantia nigra	Mean		Statistic	1,5857873
			Std. Error	,10006223
	95% Confidence Interval for	Lower Bound	Statistic	1,3804766

Descriptives

				Status (0=NC,1=ADI)
				LC
	Mean	Upper Bound	Statistic	1,8453265
	5% Trimmed Mean		Statistic	1,5690813
	Median		Statistic	1,5653103
	Variance		Statistic	,242
	Std. Deviation		Statistic	,49240262
	Minimum		Statistic	,16376
	Maximum		Statistic	2,24215
	Range		Statistic	2,07840
	Interquartile Range		Statistic	,34753
	Skewness		Statistic	-1,958
			Std. Error	,637
	Kurtosis		Statistic	6,109
			Std. Error	1,232
Midbrain	Mean		Statistic	1,6472158
			Std. Error	,14332276
	95% Confidence Interval for Mean	Lower Bound	Statistic	1,3317645
		Upper Bound	Statistic	1,9626670
	5% Trimmed Mean		Statistic	1,6928183
	Median		Statistic	1,7426180
	Variance		Statistic	,246
	Std. Deviation		Statistic	,49648462
	Minimum		Statistic	,21833
	Maximum		Statistic	2,25525
	Range		Statistic	2,03692
	Interquartile Range		Statistic	,35763
	Skewness		Statistic	-2,354
			Std. Error	,637
	Kurtosis		Statistic	7,176
			Std. Error	1,232
Pons	Mean		Statistic	,6308134
			Std. Error	,06177144
	95% Confidence Interval for	Lower Bound	Statistic	,4948554

Descriptives

				Status (0=NC,1=ADI) OB
	Mean	Upper Bound	Statistic	1,7910980
	5% Trimmed Mean		Statistic	1,5846489
	Median		Statistic	1,5626218
	Variance		Statistic	,280
	Std. Deviation		Statistic	,52947953
	Minimum		Statistic	,51010
	Maximum		Statistic	2,69618
	Range		Statistic	2,18608
	Interquartile Range		Statistic	,64465
	Skewness		Statistic	,132
			Std. Error	,441
	Kurtosis		Statistic	,177
			Std. Error	,858
Midbrain	Mean		Statistic	1,7189910
			Std. Error	,10201645
	95% Confidence Interval for Mean	Lower Bound	Statistic	1,5096705
		Upper Bound	Statistic	1,9283115
	5% Trimmed Mean		Statistic	1,7284657
	Median		Statistic	1,7529075
	Variance		Statistic	,291
	Std. Deviation		Statistic	,53982033
	Minimum		Statistic	,51522
	Maximum		Statistic	2,75144
	Range		Statistic	2,23622
	Interquartile Range		Statistic	,51834
	Skewness		Statistic	-,235
			Std. Error	,441
	Kurtosis		Statistic	,415
			Std. Error	,858
Pons	Mean		Statistic	,5916751
			Std. Error	,04348302
	95% Confidence Interval for	Lower Bound	Statistic	,5024553

Descriptives

			Status (0=NC,1=ADI)
			LC
Mean	Upper Bound	Statistic	,7667714
5% Trimmed Mean		Statistic	,6423476
Median		Statistic	,6415695
Variance		Statistic	,046
Std. Deviation		Statistic	,21398253
Minimum		Statistic	,09140
Maximum		Statistic	,96261
Range		Statistic	,87120
Interquartile Range		Statistic	,20489
Skewness		Statistic	-1,201
		Std. Error	,637
Kurtosis		Statistic	3,475
		Std. Error	1,232

Descriptives

			Status (0=NC,1=ADI)
			OB
Mean	Upper Bound	Statistic	,6808949
5% Trimmed Mean		Statistic	,5958332
Median		Statistic	,6353165
Variance		Statistic	,053
Std. Deviation		Statistic	,23009051
Minimum		Statistic	,11932
Maximum		Statistic	1,03303
Range		Statistic	,91371
Interquartile Range		Statistic	,29222
Skewness		Statistic	-,630
		Std. Error	,441
Kurtosis		Statistic	,068
		Std. Error	,858

Tests of Normality

Status (0=NC,1=ADI)		Kolmogorov-Smirnov[a] Statistic	df	Sig.	Shapiro-Wilk Statistic	df
LC	Age-BS	,135	12	,200*	,968	12
	BMI-BS	,251	12	,035	,848	12
	Beck Depression Inventory	,392	12	,000	,640	12
	SCL_90_tANX	,272	12	,014	,826	12
	BAS_Drive	,328	12	,001	,829	12
	BAS_FUN	,235	12	,066	,931	12
	BAS_Reward	,240	12	,056	,930	12
	BIS	,194	12	,200*	,895	12
	ACTH1500	,486	12	,000	,471	12
	ACTH1530_postCRH	,142	12	,200*	,928	12
	ACTH1545	,190	12	,200*	,835	12
	ACTH1600	,222	12	,107	,891	12
	ACTH1615	,218	12	,120	,871	12
	CRT1500	,258	12	,026	,748	12
	CRT1530_postCRH	,151	12	,200*	,899	12
	CRT1545	,215	12	,133	,902	12
	CRT1600	,202	12	,189	,880	12
	CRT1615	,170	12	,200*	,917	12
	Stress-ACTH-max	,210	12	,152	,917	12
	Stress-ACTH-delta-max	,200	12	,200*	,914	12
	Stress-A-AUC	,194	12	,200*	,899	12
	Stress-C-max	,154	12	,200*	,915	12
	Stress-C-dmax	,215	12	,132	,884	12
	Stress-C-AUC	,211	12	,146	,856	12
	AC_ratio_postCRH_1530	,154	12	,200*	,914	12
	AC_ratio_MAX	,158	12	,200*	,922	12
	AC_ratio_AUC	,207	12	,164	,945	12
	VOI-BS_NUK_Group	,170	12	,200*	,893	12
	Frontal cortex	,162	12	,200*	,892	12
	Orbitofronta cortex	,156	12	,200*	,920	12
	Dorsolateral prefrontal cortex	,186	12	,200*	,931	12
	Anterior cingulate cortex	,290	12	,006	,785	12
	Insula	,188	12	,200*	,870	12
	Hippocampus	,251	12	,036	,879	12
	Amygdala	,250	12	,036	,805	12

Tests of Normality

Status (0=NC,1=ADI)		Shapiro-... Sig.
LC	Age-BS	,884
	BMI-BS	,035
	Beck Depression Inventory	,000
	SCL_90_tANX	,019
	BAS_Drive	,020
	BAS_FUN	,393
	BAS_Reward	,377
	BIS	,137
	ACTH1500	,000
	ACTH1530_postCRH	,362
	ACTH1545	,024
	ACTH1600	,121
	ACTH1615	,067
	CRT1500	,003
	CRT1530_postCRH	,155
	CRT1545	,166
	CRT1600	,088
	CRT1615	,261
	Stress-ACTH-max	,263
	Stress-ACTH-delta-max	,239
	Stress-A-AUC	,153
	Stress-C-max	,247
	Stress-C-dmax	,097
	Stress-C-AUC	,044
	AC_ratio_postCRH_1530	,242
	AC_ratio_MAX	,302
	AC_ratio_AUC	,571
	VOI-BS_NUK_Group	,131
	Frontal cortex	,126
	Orbitofronta cortex	,287
	Dorsolateral prefrontal cortex	,388
	Anterior cingulate cortex	,006
	Insula	,066
	Hippocampus	,085
	Amygdala	,011

Tests of Normality

Status (0=NC,1=ADI)		Kolmogorov-Smirnov[a]			Shapiro-Wilk	
		Statistic	df	Sig.	Statistic	df
	Ncl. accumbens	,268	12	,017	,713	12
	Head of the caudate	,172	12	,200*	,935	12
	Putamen	,215	12	,131	,870	12
	Thalamus	,165	12	,200*	,945	12
	Hypothalamus	,269	12	,017	,835	12
	Substantia nigra	,306	12	,003	,785	12
	Midbrain	,271	12	,015	,739	12
	Pons	,246	12	,043	,889	12
OB	Age-BS	,132	28	,200*	,956	28
	BMI-BS	,156	28	,081	,904	28
	Beck Depression Inventory	,103	28	,200*	,958	28
	SCL_90_tANX	,153	20	,200*	,923	20
	BAS_Drive	,156	28	,080	,915	28
	BAS_FUN	,170	28	,037	,943	28
	BAS_Reward	,157	28	,075	,954	28
	BIS	,147	28	,127	,959	28
	ACTH1500	,376	28	,000	,494	28
	ACTH1530_postCRH	,225	28	,001	,674	28
	ACTH1545	,130	28	,200*	,925	28
	ACTH1600	,119	28	,200*	,903	28
	ACTH1615	,100	28	,200*	,945	28
	CRT1500	,200	28	,006	,879	28
	CRT1530_postCRH	,259	28	,000	,793	28
	CRT1545	,251	28	,000	,799	28
	CRT1600	,223	28	,001	,834	28
	CRT1615	,217	28	,002	,815	28
	Stress-ACTH-max	,219	28	,001	,731	28
	Stress-ACTH-delta-max	,203	28	,005	,763	28
	Stress-A-AUC	,107	28	,200*	,943	28
	Stress-C-max	,219	28	,001	,838	28
	Stress-C-dmax	,240	28	,000	,821	28
	Stress-C-AUC	,233	28	,000	,825	28
	AC_ratio_postCRH_1530	,340	28	,000	,366	28
	AC_ratio_MAX	,352	28	,000	,374	28
	AC_ratio_AUC	,221	28	,001	,704	28
	VOI-BS_NUK_Group	,091	28	,200*	,946	28

Tests of Normality

Status (0=NC,1=ADI)		Shapiro-... Sig.
	Ncl. accumbens	,001
	Head of the caudate	,437
	Putamen	,065
	Thalamus	,571
	Hypothalamus	,024
	Substantia nigra	,006
	Midbrain	,002
	Pons	,113
OB	Age-BS	,278
	BMI-BS	,014
	Beck Depression Inventory	,320
	SCL_90_tANX	,114
	BAS_Drive	,026
	BAS_FUN	,129
	BAS_Reward	,246
	BIS	,338
	ACTH1500	,000
	ACTH1530_postCRH	,000
	ACTH1545	,045
	ACTH1600	,014
	ACTH1615	,146
	CRT1500	,004
	CRT1530_postCRH	,000
	CRT1545	,000
	CRT1600	,000
	CRT1615	,000
	Stress-ACTH-max	,000
	Stress-ACTH-delta-max	,000
	Stress-A-AUC	,132
	Stress-C-max	,001
	Stress-C-dmax	,000
	Stress-C-AUC	,000
	AC_ratio_postCRH_1530	,000
	AC_ratio_MAX	,000
	AC_ratio_AUC	,000
	VOI-BS_NUK_Group	,158

Tests of Normality

Status (0=NC,1=ADI)		Kolmogorov-Smirnov[a]			Shapiro-Wilk	
		Statistic	df	Sig.	Statistic	df
	Frontal cortex	,145	28	,134	,958	28
	Orbitofronta cortex	,098	28	,200*	,960	28
	Dorsolateral prefrontal cortex	,140	28	,167	,965	28
	Anterior cingulate cortex	,067	28	,200*	,975	28
	Insula	,106	28	,200*	,963	28
	Hippocampus	,149	28	,114	,864	28
	Amygdala	,167	28	,044	,928	28
	Ncl. accumbens	,120	28	,200*	,965	28
	Head of the caudate	,145	28	,139	,952	28
	Putamen	,070	28	,200*	,984	28
	Thalamus	,119	28	,200*	,957	28
	Hypothalamus	,124	28	,200*	,919	28
	Substantia nigra	,147	28	,125	,968	28
	Midbrain	,118	28	,200*	,960	28
	Pons	,158	28	,071	,939	28

Tests of Normality

Status (0=NC,1=ADI)		Shapiro-...
		Sig.
	Frontal cortex	,318
	Orbitofronta cortex	,350
	Dorsolateral prefrontal cortex	,451
	Anterior cingulate cortex	,706
	Insula	,410
	Hippocampus	,002
	Amygdala	,055
	Ncl. accumbens	,454
	Head of the caudate	,228
	Putamen	,939
	Thalamus	,294
	Hypothalamus	,033
	Substantia nigra	,536
	Midbrain	,352
	Pons	,107

*. This is a lower bound of the true significance.

a. Lilliefors Significance Correction

```
FREQUENCIES VARIABLES=Sex
  /ORDER=ANALYSIS.
```

Frequencies

Notes

Output Created		21-SEP-2022 00:01:23
Comments		
Input	Data	C:\Users\Christian Schinke\OneDrive - Charité - Universitätsmedizin Berlin\Promotion und Projekte Leipzig\02_DASB vs. HPA\06_Open_Data\01_Data_public.sav
	Active Dataset	DataSet1
	Filter	NOT INCLUSION_EXCLUSION =9 (FILTER)
	Weight	<none>
	Split File	Status (0=NC,1=ADI)
	N of Rows in Working Data File	40
Missing Value Handling	Definition of Missing	User-defined missing values are treated as missing.
	Cases Used	Statistics are based on all cases with valid data.
Syntax		FREQUENCIES VARIABLES=Sex /ORDER=ANALYSIS.
Resources	Processor Time	00:00:00,00
	Elapsed Time	00:00:00,00

Statistics

Gender (0=female,1=male)

LC	N	Valid	12
		Missing	0
OB	N	Valid	28
		Missing	0

Gender (0=female,1=male)

Status (0=NC,1=ADI)			Frequency	Percent	Valid Percent	Cumulative Percent
LC	Valid	female	8	66,7	66,7	66,7
		male	4	33,3	33,3	100,0
		Total	12	100,0	100,0	
OB	Valid	female	21	75,0	75,0	75,0
		male	7	25,0	25,0	100,0
		Total	28	100,0	100,0	

```
FREQUENCIES VARIABLES=Age BMI_Baseline_PET BDI SCL_90_tANX BAS_Drive BAS_FU
N BAS_Reward BIS
  /FORMAT=NOTABLE
  /NTILES=4
  /STATISTICS=STDDEV MEAN MEDIAN
  /ORDER=ANALYSIS.
```

Frequencies

Notes

Output Created		21-SEP-2022 00:01:23
Comments		
Input	Data	C:\Users\Christian Schinke\OneDrive - Charité - Universitätsmedizin Berlin\Promotion und Projekte Leipzig\02_DASB vs. HPA\06_Open_Data\01_Data_public.sav
	Active Dataset	DataSet1
	Filter	NOT INCLUSION_EXCLUSION =9 (FILTER)
	Weight	<none>
	Split File	Status (0=NC,1=ADI)
	N of Rows in Working Data File	40
Missing Value Handling	Definition of Missing	User-defined missing values are treated as missing.
	Cases Used	Statistics are based on all cases with valid data.
Syntax		FREQUENCIES VARIABLES=Age BMI_Baseline_PET BDI SCL_90_tANX BAS_Drive BAS_FUN BAS_Reward BIS<br>/FORMAT=NOTABLE<br>/NTILES=4<br>/STATISTICS=STDDEV MEAN MEDIAN<br>/ORDER=ANALYSIS.
Resources	Processor Time	00:00:00,00
	Elapsed Time	00:00:00,00

Statistics

		Status (0=NC,1=ADI)				
		LC				
		Age-BS	BMI-BS	Beck Depression Inventory	SCL_90_tANX	BAS_Drive
N	Valid	12	12	12	12	12
	Missing	0	0	0	0	0
Mean		35,75	22,401	1,1667	44,7500	13,1667
Median		36,00	22,440	,0000	45,0000	14,0000
Std. Deviation		7,362	2,2646	1,94625	5,69090	1,26730
Percentiles	25	30,00	20,085	,0000	38,7500	12,0000
	50	36,00	22,440	,0000	45,0000	14,0000
	75	40,00	24,699	3,2500	49,0000	14,0000

Statistics

		Status (0=NC,1=ADI)				
		LC			OB	
		BAS_FUN	BAS_Reward	BIS	Age-BS	BMI-BS
N	Valid	12	12	12	28	28
	Missing	0	0	0	0	0
Mean		12,4167	17,25	18,5833	36,57	41,152
Median		12,5000	18,00	19,0000	35,00	39,801
Std. Deviation		1,67649	1,865	2,67848	10,570	5,0905
Percentiles	25	12,0000	15,25	17,0000	28,50	36,605
	50	12,5000	18,00	19,0000	35,00	39,801
	75	13,7500	18,75	20,7500	43,50	44,572

Statistics

		Status (0=NC,1=ADI) OB				
		Beck Depression Inventory	SCL_90_tANX	BAS_Drive	BAS_FUN	BAS_Reward
N	Valid	28	20	28	28	28
	Missing	0	8	0	0	0
Mean		7,2500	48,9500	12,5000	11,0714	16,31
Median		6,5000	49,0000	13,0000	11,0000	16,50
Std. Deviation		4,91878	7,59137	2,09938	1,98006	2,172
Percentiles	25	3,0000	41,0000	11,2500	10,0000	15,00
	50	6,5000	49,0000	13,0000	11,0000	16,50
	75	11,0000	52,0000	14,0000	12,0000	18,00

Statistics

		Status (0=NC,... OB
		BIS
N	Valid	28
	Missing	0
Mean		19,0357
Median		19,0000
Std. Deviation		3,76615
Percentiles	25	17,0000
	50	19,0000
	75	22,0000

```
FREQUENCIES VARIABLES=ACTH1500 ACTH1530_postCRH ACTH_MAX ACTH_AUC CRT1500 C
RT1530_postCRH CRT_MAX
    CRT_AUC AC_ratio_postCRH1530 AC_ratio_MAX AC_ratio_AUC
  /FORMAT=NOTABLE
  /NTILES=4
  /STATISTICS=STDDEV MEAN MEDIAN
  /ORDER=ANALYSIS.
```

Frequencies

Notes

Output Created		21-SEP-2022 00:01:23
Comments		
Input	Data	C:\Users\Christian Schinke\OneDrive - Charité - Universitätsmedizin Berlin\Promotion und Projekte Leipzig\02_DASB vs. HPA\06_Open_Data\01_Data_public.sav
	Active Dataset	DataSet1
	Filter	NOT INCLUSION_EXCLUSION=9 (FILTER)
	Weight	<none>
	Split File	Status (0=NC,1=ADI)
	N of Rows in Working Data File	40
Missing Value Handling	Definition of Missing	User-defined missing values are treated as missing.
	Cases Used	Statistics are based on all cases with valid data.
Syntax		FREQUENCIES VARIABLES=ACTH1500 ACTH1530_postCRH ACTH_MAX ACTH_AUC CRT1500 CRT1530_postCRH CRT_MAX CRT_AUC AC_ratio_postCRH_1530 AC_ratio_MAX AC_ratio_AUC /FORMAT=NOTABLE /NTILES=4 /STATISTICS=STDDEV MEAN MEDIAN /ORDER=ANALYSIS.
Resources	Processor Time	00:00:00,00
	Elapsed Time	00:00:00,00

Statistics

			Status (0=NC,1=ADI) LC	OB
ACTH1500	N	Valid	12	28
		Missing	0	0
	Mean		,8575	,8982
	Median		,8300	,8300
	Std. Deviation		,06995	,16382
	Percentiles	25	,8300	,8300
		50	,8300	,8300
		75	,8300	,8675
ACTH1530_postCRH	N	Valid	12	28
		Missing	0	0
	Mean		1,4800	2,1182
	Median		1,4750	1,6600
	Std. Deviation		,55120	1,60524
	Percentiles	25	,9100	1,2100
		50	1,4750	1,6600
		75	1,8225	2,4225
Stress-ACTH-max	N	Valid	12	28
		Missing	0	0
	Mean		2,1708	2,5079
	Median		1,8500	2,1050
	Std. Deviation		1,12199	1,56314
	Percentiles	25	1,3425	1,5725
		50	1,8500	2,1050
		75	3,0625	2,9750
Stress-A-AUC	N	Valid	12	28
		Missing	0	0
	Mean		6,51083	7,27339
	Median		5,52750	6,77500
	Std. Deviation		2,908188	2,738694
	Percentiles	25	4,05375	5,03250
		50	5,52750	6,77500
		75	8,81875	9,20625
CRT1500	N	Valid	12	28
		Missing	0	0
	Mean		21,3583	20,7571
	Median		15,8000	19,9500
	Std. Deviation		17,61324	8,51884

Statistics

			Status (0=NC,1=ADI) LC	OB
	Percentiles	25	10,9500	15,1750
		50	15,8000	19,9500
		75	24,2250	23,2750
CRT1530_postCRH	N	Valid	12	28
		Missing	0	0
	Mean		29,5333	98,8393
	Median		25,5000	48,9000
	Std. Deviation		14,85257	100,86071
	Percentiles	25	20,0500	32,5500
		50	25,5000	48,9000
		75	38,0500	154,1250
Stress-C-max	N	Valid	12	28
		Missing	0	0
	Mean		67,2333	121,9536
	Median		66,3500	74,3500
	Std. Deviation		41,80307	113,71667
	Percentiles	25	25,8750	38,5500
		50	66,3500	74,3500
		75	100,7000	170,5250
Stress-C-AUC	N	Valid	12	28
		Missing	0	0
	Mean		171,1958	372,8250
	Median		165,3750	216,0000
	Std. Deviation		87,75002	351,63457
	Percentiles	25	87,7750	114,1500
		50	165,3750	216,0000
		75	262,4500	525,4500
AC_ratio_postCRH_1530	N	Valid	12	28
		Missing	0	0
	Mean		,06126217	,05621411
	Median		,05893849	,02850999
	Std. Deviation		,033288788	,117879256
	Percentiles	25	,03423931	,01292332
		50	,05893849	,02850999
		75	,09736015	,04993133
AC_ratio_MAX	N	Valid	12	28
		Missing	0	0

Statistics

			Status (0=NC,1=ADI)	
			LC	OB
	Mean		,039807	,055489
	Median		,037810	,023225
	Std. Deviation		,0201296	,1167906
	Percentiles	25	,021223	,013178
		50	,037810	,023225
		75	,061985	,047158
AC_ratio_AUC	N	Valid	12	28
		Missing	0	0
	Mean		,045963	,041641
	Median		,042860	,025295
	Std. Deviation		,0234335	,0442723
	Percentiles	25	,027048	,014485
		50	,042860	,025295
		75	,065870	,053173

```
SPLIT FILE OFF.

T-TEST GROUPS=Status_0_NO_1_OB(0 1)
  /MISSING=ANALYSIS
  /VARIABLES=Age BMI_Baseline_PET
  /CRITERIA=CI(.95).
```

T-Test

Notes

Output Created		21-SEP-2022 00:01:23
Comments		
Input	Data	C:\Users\Christian Schinke\OneDrive - Charité - Universitätsmedizin Berlin\Promotion und Projekte Leipzig\02_DASB vs. HPA\06_Open_Data\01_Data_public.sav
	Active Dataset	DataSet1
	Filter	NOT INCLUSION_EXCLUSION=9 (FILTER)
	Weight	<none>
	Split File	<none>
	N of Rows in Working Data File	40
Missing Value Handling	Definition of Missing	User defined missing values are treated as missing.
	Cases Used	Statistics for each analysis are based on the cases with no missing or out-of-range data for any variable in the analysis.
Syntax		T-TEST GROUPS=Status_0_NO_1_OB(0 1)<br>/MISSING=ANALYSIS<br>/VARIABLES=Age BMI_Baseline_PET<br>/CRITERIA=CI(.95).
Resources	Processor Time	00:00:00,00
	Elapsed Time	00:00:00,00

Group Statistics

	Status (0=NC,1=ADI)	N	Mean	Std. Deviation	Std. Error Mean
Age-BS	LC	12	35,75	7,362	2,125
	OB	28	36,57	10,570	1,998
BMI-BS	LC	12	22,401	2,2646	,6537
	OB	28	41,152	5,0905	,9620

Independent Samples Test

		Levene's Test for Equality of Variances		t-test for Equality of Means	
		F	Sig.	t	df
Age-BS	Equal variances assumed	2,486	,123	-,244	38
	Equal variances not assumed			-,282	29,607
BMI-BS	Equal variances assumed	8,052	,007	-12,184	38
	Equal variances not assumed			-16,122	37,871

Independent Samples Test

		t-test for Equality of Means		
		Sig. (2-tailed)	Mean Difference	Std. Error Difference
Age-BS	Equal variances assumed	,808	-,821	3,364
	Equal variances not assumed	,780	-,821	2,917
BMI-BS	Equal variances assumed	,000	-18,7512	1,5390
	Equal variances not assumed	,000	-18,7512	1,1631

Independent Samples Test

		t-test for Equality of Means	
		95% Confidence Interval of the Difference	
		Lower	Upper
Age-BS	Equal variances assumed	-7,632	5,989
	Equal variances not assumed	-6,782	5,139
BMI-BS	Equal variances assumed	-21,8669	-15,6356
	Equal variances not assumed	-21,1061	-16,3964

```
CROSSTABS
  /TABLES=Status_0_NO_1_OB BY Sex
  /FORMAT=AVALUE TABLES
  /STATISTICS=CHISQ
  /CELLS=COUNT
  /COUNT ROUND CELL.
```

Crosstabs

Notes

Output Created		21-SEP-2022 00:01:23
Comments		
Input	Data	C:\Users\Christian Schinke\OneDrive - Charité - Universitätsmedizin Berlin\Promotion und Projekte Leipzig\02_DASB vs. HPA\06_Open_Data\01_Data_public.sav
	Active Dataset	DataSet1
	Filter	NOT INCLUSION_EXCLUSION =9 (FILTER)
	Weight	<none>
	Split File	<none>
	N of Rows in Working Data File	40
Missing Value Handling	Definition of Missing	User-defined missing values are treated as missing.
	Cases Used	Statistics for each table are based on all the cases with valid data in the specified range(s) for all variables in each table.
Syntax		CROSSTABS /TABLES=Status_0_NO_1_OB BY Sex /FORMAT=AVALUE TABLES /STATISTICS=CHISQ /CELLS=COUNT /COUNT ROUND CELL.
Resources	Processor Time	00:00:00,00
	Elapsed Time	00:00:00,00
	Dimensions Requested	2
	Cells Available	524245

Case Processing Summary

	Cases					
	Valid		Missing		Total	
	N	Percent	N	Percent	N	Percent
Status (0=NC,1=ADI) * Gender (0=female,1=male)	40	100,0%	0	0,0%	40	100,0%

Status (0=NC,1=ADI) * Gender (0=female,1=male) Crosstabulation

Count

		Gender (0=female,1=male)		Total
		female	male	
Status (0=NC,1=ADI)	LC	8	4	12
	OB	21	7	28
Total		29	11	40

Chi-Square Tests

	Value	df	Asymptotic Significance (2-sided)	Exact Sig. (2-sided)	Exact Sig. (1-sided)
Pearson Chi-Square	,293[a]	1	,589		
Continuity Correction[b]	,024	1	,877		
Likelihood Ratio	,286	1	,593		
Fisher's Exact Test				,704	,430
Linear-by-Linear Association	,285	1	,593		
N of Valid Cases	40				

a. 1 cells (25,0%) have expected count less than 5. The minimum expected count is 3,30.

b. Computed only for a 2x2 table

```
SPLIT FILE OFF.

NPAR TESTS
  /M-W= BDI SCL_90_tANX BAS_Drive BAS_FUN BAS_Reward BIS BY Status_0_NO_1_O
B(0 1)
  /MISSING ANALYSIS.
```

NPar Tests

Notes

Output Created		21-SEP-2022 00:01:23
Comments		
Input	Data	C:\Users\Christian Schinke\OneDrive - Charité - Universitätsmedizin Berlin\Promotion und Projekte Leipzig\02_DASB vs. HPA\06_Open_Data\01_Data_public.sav
	Active Dataset	DataSet1
	Filter	NOT INCLUSION_EXCLUSION =9 (FILTER)
	Weight	<none>
	Split File	<none>
	N of Rows in Working Data File	40
Missing Value Handling	Definition of Missing	User-defined missing values are treated as missing.
	Cases Used	Statistics for each test are based on all cases with valid data for the variable (s) used in that test.
Syntax		NPAR TESTS /M-W= BDI SCL_90_tANX BAS_Drive BAS_FUN BAS_Reward BIS BY Status_0_NO_1_OB(0 1) /MISSING ANALYSIS.
Resources	Processor Time	00:00:00,00
	Elapsed Time	00:00:00,00
	Number of Cases Allowed[a]	262144

a. Based on availability of workspace memory.

Mann-Whitney Test

Ranks

	Status (0=NC,1=ADI)	N	Mean Rank	Sum of Ranks
Beck Depression Inventory	LC	12	9,75	117,00
	OB	28	25,11	703,00
	Total	40		
SCL_90_tANX	LC	12	12,92	155,00
	OB	20	18,65	373,00
	Total	32		
BAS_Drive	LC	12	22,46	269,50
	OB	28	19,66	550,50
	Total	40		
BAS_FUN	LC	12	26,58	319,00
	OB	28	17,89	501,00
	Total	40		
BAS_Reward	LC	12	23,96	287,50
	OB	28	19,02	532,50
	Total	40		
BIS	LC	12	19,96	239,50
	OB	28	20,73	580,50
	Total	40		

Test Statistics[a]

	Beck Depression Inventory	SCL_90_tANX	BAS_Drive	BAS_FUN	BAS_Reward
Mann-Whitney U	39,000	77,000	144,500	95,000	126,500
Wilcoxon W	117,000	155,000	550,500	501,000	532,500
Z	-3,843	-1,707	-,711	-2,191	-1,240
Asymp. Sig. (2-tailed)	,000	,088	,477	,028	,215
Exact Sig. [2*(1-tailed Sig.)]	,000[b]	,099[b]	,493[b]	,031[b]	,224[b]

Test Statistics[a]

	BIS
Mann-Whitney U	161,500
Wilcoxon W	239,500
Z	-,193
Asymp. Sig. (2-tailed)	,847
Exact Sig. [2*(1-tailed Sig.)]	,850[b]

a. Grouping Variable: Status (0=NC,1=ADI)

b. Not corrected for ties.

```
NPAR TESTS
 /M-W= ACTH1500 ACTH1530_postCRH ACTH_MAX ACTH_AUC CRT1500 CRT1530_postCRH
CRT_MAX
   CRT_AUC AC_ratio_postCRH1530 AC_ratio_MAX AC_ratio_AUC BY Status_0_NO_
1_OB(0 1)
 /MISSING ANALYSIS.
```

NPar Tests

Notes

Output Created		21-SEP-2022 00:01:23
Comments		
Input	Data	C:\Users\Christian Schinke\OneDrive - Charité - Universitätsmedizin Berlin\Promotion und Projekte Leipzig\02_DASB vs. HPA\06_Open_Data\01_Data_public.sav
	Active Dataset	DataSet1
	Filter	NOT INCLUSION_EXCLUSION =9 (FILTER)
	Weight	<none>
	Split File	<none>
	N of Rows in Working Data File	40
Missing Value Handling	Definition of Missing	User-defined missing values are treated as missing.
	Cases Used	Statistics for each test are based on all cases with valid data for the variable (s) used in that test.

Notes

Syntax		NPAR TESTS<br>/M-W= ACTH1500<br>ACTH1530_postCRH<br>ACTH_MAX ACTH_AUC<br>CRT1500<br>CRT1530_postCRH<br>CRT_MAX<br>CRT_AUC<br>AC_ratio_postCRH_1530<br>AC_ratio_MAX<br>AC_ratio_AUC BY<br>Status_0_NO_1_OB(0 1)<br>/MISSING ANALYSIS.
Resources	Processor Time	00:00:00,00
	Elapsed Time	00:00:00,00
	Number of Cases Allowed[a]	185042

a. Based on availability of workspace memory.

Mann-Whitney Test

Ranks

	Status (0=NC,1=ADI)	N	Mean Rank	Sum of Ranks
ACTH1500	LC	12	18,79	225,50
	OB	28	21,23	594,50
	Total	40		
ACTH1530_postCRH	LC	12	16,67	200,00
	OB	28	22,14	620,00
	Total	40		
Stress-ACTH-max	LC	12	18,42	221,00
	OB	28	21,39	599,00
	Total	40		
Stress-A-AUC	LC	12	17,75	213,00
	OB	28	21,68	607,00
	Total	40		
CRT1500	LC	12	17,50	210,00
	OB	28	21,79	610,00
	Total	40		
CRT1530_postCRH	LC	12	13,58	163,00
	OB	28	23,46	657,00
	Total	40		

Ranks

	Status (0=NC,1=ADI)	N	Mean Rank	Sum of Ranks
Stress-C-max	LC	12	17,50	210,00
	OB	28	21,79	610,00
	Total	40		
Stress-C-AUC	LC	12	17,00	204,00
	OB	28	22,00	616,00
	Total	40		
AC_ratio_postCRH_1530	LC	12	27,00	324,00
	OB	28	17,71	496,00
	Total	40		
AC_ratio_MAX	LC	12	24,00	288,00
	OB	28	19,00	532,00
	Total	40		
AC_ratio_AUC	LC	12	24,17	290,00
	OB	28	18,93	530,00
	Total	40		

Test Statistics[a]

	ACTH1500	ACTH1530_postCRH	Stress-ACTH-max	Stress-A-AUC	CRT1500
Mann-Whitney U	147,500	122,000	143,000	135,000	132,000
Wilcoxon W	225,500	200,000	221,000	213,000	210,000
Z	-,796	-1,359	-,738	-,974	-1,063
Asymp. Sig. (2-tailed)	,426	,174	,461	,330	,288
Exact Sig. [2*(1-tailed Sig.)]	,550[b]	,182[b]	,475[b]	,342[b]	,299[b]

Test Statistics[a]

	CRT1530_postCRH	Stress-C-max	Stress-C-AUC	AC_ratio_postCRH_1530
Mann-Whitney U	85,000	132,000	126,000	90,000
Wilcoxon W	163,000	210,000	204,000	496,000
Z	-2,450	-1,063	-1,240	-2,302
Asymp. Sig. (2-tailed)	,014	,288	,215	,021
Exact Sig. [2*(1-tailed Sig.)]	,013[b]	,299[b]	,224[b]	,021[b]

Test Statistics[a]

	AC_ratio_MAX	AC_ratio_AUC
Mann-Whitney U	126,000	124,000
Wilcoxon W	532,000	530,000
Z	-1,240	-1,299
Asymp. Sig. (2-tailed)	,215	,194
Exact Sig. [2*(1-tailed Sig.)]	,224[b]	,202[b]

a. Grouping Variable: Status (0=NC,1=ADI)

b. Not corrected for ties.

```
 SORT CASES  BY Status_0_NO_1_OB.
SPLIT FILE SEPARATE BY Status_0_NO_1_OB.

NONPAR CORR
  /VARIABLES=ACTH_MAX ACTH_AUC CRT_MAX CRT_AUC SERT_BPND_Group_averageFC O
FC DLPFC ACC INS HI AMG
    NAcc CD PU TH HYPOTH SN_VTA MIB PONS
  /PRINT=SPEARMAN TWOTAIL NOSIG
  /MISSING=PAIRWISE.
```

Nonparametric Correlations

Notes

Output Created		21-SEP-2022 00:01:23
Comments		
Input	Data	C:\Users\Christian Schinke\OneDrive - Charité - Universitätsmedizin Berlin\Promotion und Projekte Leipzig\02_DASB vs. HPA\06_Open_Data\01_Data_public.sav
	Active Dataset	DataSet1
	Filter	NOT INCLUSION_EXCLUSION =9 (FILTER)
	Weight	<none>
	Split File	Status (0=NC,1=ADI)
	N of Rows in Working Data File	40
Missing Value Handling	Definition of Missing	User-defined missing values are treated as missing.
	Cases Used	Statistics for each pair of variables are based on all the cases with valid data for that pair.
Syntax		NONPAR CORR /VARIABLES=ACTH_MAX ACTH_AUC CRT_MAX CRT_AUC SERT_BPND_Group_average FC OFC DLPFC ACC INS HI AMG NAcc CD PU TH HYPOTH SN_VTA MIB PONS /PRINT=SPEARMAN TWOTAIL NOSIG...
Resources	Processor Time	00:00:00,02
	Elapsed Time	00:00:00,03
	Number of Cases Allowed	136770 cases[a]

a. Based on availability of workspace memory

Status (0=NC,1=ADI) = LC

Correlations[a]

			Stress-ACTH-max	Stress-A-AUC
Spearman's rho	Stress-ACTH-max	Correlation Coefficient	1,000	,944**
		Sig. (2-tailed)	.	,000
		N	12	12
	Stress-A-AUC	Correlation Coefficient	,944**	1,000
		Sig. (2-tailed)	,000	.
		N	12	12
	Stress-C-max	Correlation Coefficient	,566	,524
		Sig. (2-tailed)	,055	,080
		N	12	12
	Stress-C-AUC	Correlation Coefficient	,413	,336
		Sig. (2-tailed)	,183	,286
		N	12	12
	VOI-BS_NUK_Group	Correlation Coefficient	-,147	,049
		Sig. (2-tailed)	,649	,880
		N	12	12
	Frontal cortex	Correlation Coefficient	,147	,231
		Sig. (2-tailed)	,649	,471
		N	12	12
	Orbitofronta cortex	Correlation Coefficient	,007	,091
		Sig. (2-tailed)	,983	,779
		N	12	12
	Dorsolateral prefrontal cortex	Correlation Coefficient	,091	,133
		Sig. (2-tailed)	,779	,681
		N	12	12
	Anterior cingulate cortex	Correlation Coefficient	-,301	-,168
		Sig. (2-tailed)	,342	,602
		N	12	12
	Insula	Correlation Coefficient	,161	,231
		Sig. (2-tailed)	,618	,471
		N	12	12
	Hippocampus	Correlation Coefficient	,524	,545
		Sig. (2-tailed)	,080	,067
		N	12	12
	Amygdala	Correlation Coefficient	-,399	-,378
		Sig. (2-tailed)	,199	,226
		N	12	12

Correlations[a]

			Stress-C-max	Stress-C-AUC
Spearman's rho	Stress-ACTH-max	Correlation Coefficient	,566	,413
		Sig. (2-tailed)	,055	,183
		N	12	12
	Stress-A-AUC	Correlation Coefficient	,524	,336
		Sig. (2-tailed)	,080	,286
		N	12	12
	Stress-C-max	Correlation Coefficient	1,000	,916**
		Sig. (2-tailed)	.	,000
		N	12	12
	Stress-C-AUC	Correlation Coefficient	,916**	1,000
		Sig. (2-tailed)	,000	.
		N	12	12
	VOI-BS_NUK_Group	Correlation Coefficient	,084	,133
		Sig. (2-tailed)	,795	,681
		N	12	12
	Frontal cortex	Correlation Coefficient	,420	,559
		Sig. (2-tailed)	,175	,059
		N	12	12
	Orbitofronta cortex	Correlation Coefficient	,252	,077
		Sig. (2-tailed)	,430	,812
		N	12	12
	Dorsolateral prefrontal cortex	Correlation Coefficient	,455	,503
		Sig. (2-tailed)	,138	,095
		N	12	12
	Anterior cingulate cortex	Correlation Coefficient	,119	,203
		Sig. (2-tailed)	,713	,527
		N	12	12
	Insula	Correlation Coefficient	,378	,364
		Sig. (2-tailed)	,226	,245
		N	12	12
	Hippocampus	Correlation Coefficient	,524	,594*
		Sig. (2-tailed)	,080	,042
		N	12	12
	Amygdala	Correlation Coefficient	-,538	-,392
		Sig. (2-tailed)	,071	,208
		N	12	12

Correlations[a]

			VOI-BS_NUK_Group	Frontal cortex
Spearman's rho	Stress-ACTH-max	Correlation Coefficient	-,147	,147
		Sig. (2-tailed)	,649	,649
		N	12	12
	Stress-A-AUC	Correlation Coefficient	,049	,231
		Sig. (2-tailed)	,880	,471
		N	12	12
	Stress-C-max	Correlation Coefficient	,084	,420
		Sig. (2-tailed)	,795	,175
		N	12	12
	Stress-C-AUC	Correlation Coefficient	,133	,559
		Sig. (2-tailed)	,681	,059
		N	12	12
	VOI-BS_NUK_Group	Correlation Coefficient	1,000	,580*
		Sig. (2-tailed)	.	,048
		N	12	12
	Frontal cortex	Correlation Coefficient	,580*	1,000
		Sig. (2-tailed)	,048	.
		N	12	12
	Orbitofronta cortex	Correlation Coefficient	,343	,161
		Sig. (2-tailed)	,276	,618
		N	12	12
	Dorsolateral prefrontal cortex	Correlation Coefficient	,371	,797**
		Sig. (2-tailed)	,236	,002
		N	12	12
	Anterior cingulate cortex	Correlation Coefficient	,559	,559
		Sig. (2-tailed)	,059	,059
		N	12	12
	Insula	Correlation Coefficient	,503	,566
		Sig. (2-tailed)	,095	,055
		N	12	12
	Hippocampus	Correlation Coefficient	,266	,531
		Sig. (2-tailed)	,404	,075
		N	12	12
	Amygdala	Correlation Coefficient	,210	-,007
		Sig. (2-tailed)	,513	,983
		N	12	12

Correlations[a]

			Orbitofronta cortex
Spearman's rho	Stress-ACTH-max	Correlation Coefficient	,007
		Sig. (2-tailed)	,983
		N	12
	Stress-A-AUC	Correlation Coefficient	,091
		Sig. (2-tailed)	,779
		N	12
	Stress-C-max	Correlation Coefficient	,252
		Sig. (2-tailed)	,430
		N	12
	Stress-C-AUC	Correlation Coefficient	,077
		Sig. (2-tailed)	,812
		N	12
	VOI-BS_NUK_Group	Correlation Coefficient	,343
		Sig. (2-tailed)	,276
		N	12
	Frontal cortex	Correlation Coefficient	,161
		Sig. (2-tailed)	,618
		N	12
	Orbitofronta cortex	Correlation Coefficient	1,000
		Sig. (2-tailed)	.
		N	12
	Dorsolateral prefrontal cortex	Correlation Coefficient	,455
		Sig. (2-tailed)	,138
		N	12
	Anterior cingulate cortex	Correlation Coefficient	,503
		Sig. (2-tailed)	,095
		N	12
	Insula	Correlation Coefficient	,762**
		Sig. (2-tailed)	,004
		N	12
	Hippocampus	Correlation Coefficient	,028
		Sig. (2-tailed)	,931
		N	12
	Amygdala	Correlation Coefficient	,259
		Sig. (2-tailed)	,417
		N	12

Correlations[a]

			Dorsolateral prefrontal cortex
Spearman's rho	Stress-ACTH-max	Correlation Coefficient	,091
		Sig. (2-tailed)	,779
		N	12
	Stress-A-AUC	Correlation Coefficient	,133
		Sig. (2-tailed)	,681
		N	12
	Stress-C-max	Correlation Coefficient	,455
		Sig. (2-tailed)	,138
		N	12
	Stress-C-AUC	Correlation Coefficient	,503
		Sig. (2-tailed)	,095
		N	12
	VOI-BS_NUK_Group	Correlation Coefficient	,371
		Sig. (2-tailed)	,236
		N	12
	Frontal cortex	Correlation Coefficient	,797**
		Sig. (2-tailed)	,002
		N	12
	Orbitofronta cortex	Correlation Coefficient	,455
		Sig. (2-tailed)	,138
		N	12
	Dorsolateral prefrontal cortex	Correlation Coefficient	1,000
		Sig. (2-tailed)	.
		N	12
	Anterior cingulate cortex	Correlation Coefficient	,727**
		Sig. (2-tailed)	,007
		N	12
	Insula	Correlation Coefficient	,797**
		Sig. (2-tailed)	,002
		N	12
	Hippocampus	Correlation Coefficient	,510
		Sig. (2-tailed)	,090
		N	12
	Amygdala	Correlation Coefficient	,133
		Sig. (2-tailed)	,681
		N	12

Correlations[a]

			Anterior cingulate cortex	Insula
Spearman's rho	Stress-ACTH-max	Correlation Coefficient	-,301	,161
		Sig. (2-tailed)	,342	,618
		N	12	12
	Stress-A-AUC	Correlation Coefficient	-,168	,231
		Sig. (2-tailed)	,602	,471
		N	12	12
	Stress-C-max	Correlation Coefficient	,119	,378
		Sig. (2-tailed)	,713	,226
		N	12	12
	Stress-C-AUC	Correlation Coefficient	,203	,364
		Sig. (2-tailed)	,527	,245
		N	12	12
	VOI-BS_NUK_Group	Correlation Coefficient	,559	,503
		Sig. (2-tailed)	,059	,095
		N	12	12
	Frontal cortex	Correlation Coefficient	,559	,566
		Sig. (2-tailed)	,059	,055
		N	12	12
	Orbitofronta cortex	Correlation Coefficient	,503	,762**
		Sig. (2-tailed)	,095	,004
		N	12	12
	Dorsolateral prefrontal cortex	Correlation Coefficient	,727**	,797**
		Sig. (2-tailed)	,007	,002
		N	12	12
	Anterior cingulate cortex	Correlation Coefficient	1,000	,664*
		Sig. (2-tailed)	.	,018
		N	12	12
	Insula	Correlation Coefficient	,664*	1,000
		Sig. (2-tailed)	,018	.
		N	12	12
	Hippocampus	Correlation Coefficient	,112	,455
		Sig. (2-tailed)	,729	,138
		N	12	12
	Amygdala	Correlation Coefficient	,252	,280
		Sig. (2-tailed)	,430	,379
		N	12	12

Correlations[a]

			Hippocampus	Amygdala
Spearman's rho	Stress-ACTH-max	Correlation Coefficient	,524	-,399
		Sig. (2-tailed)	,080	,199
		N	12	12
	Stress-A-AUC	Correlation Coefficient	,545	-,378
		Sig. (2-tailed)	,067	,226
		N	12	12
	Stress-C-max	Correlation Coefficient	,524	-,538
		Sig. (2-tailed)	,080	,071
		N	12	12
	Stress-C-AUC	Correlation Coefficient	,594*	-,392
		Sig. (2-tailed)	,042	,208
		N	12	12
	VOI-BS_NUK_Group	Correlation Coefficient	,266	,210
		Sig. (2-tailed)	,404	,513
		N	12	12
	Frontal cortex	Correlation Coefficient	,531	-,007
		Sig. (2-tailed)	,075	,983
		N	12	12
	Orbitofronta cortex	Correlation Coefficient	,028	,259
		Sig. (2-tailed)	,931	,417
		N	12	12
	Dorsolateral prefrontal cortex	Correlation Coefficient	,510	,133
		Sig. (2-tailed)	,090	,681
		N	12	12
	Anterior cingulate cortex	Correlation Coefficient	,112	,252
		Sig. (2-tailed)	,729	,430
		N	12	12
	Insula	Correlation Coefficient	,455	,280
		Sig. (2-tailed)	,138	,379
		N	12	12
	Hippocampus	Correlation Coefficient	1,000	,175
		Sig. (2-tailed)	.	,587
		N	12	12
	Amygdala	Correlation Coefficient	,175	1,000
		Sig. (2-tailed)	,587	.
		N	12	12

Correlations[a]

			Ncl. accumbens
Spearman's rho	Stress-ACTH-max	Correlation Coefficient	-,140
		Sig. (2-tailed)	,665
		N	12
	Stress-A-AUC	Correlation Coefficient	-,287
		Sig. (2-tailed)	,366
		N	12
	Stress-C-max	Correlation Coefficient	-,091
		Sig. (2-tailed)	,779
		N	12
	Stress-C-AUC	Correlation Coefficient	,140
		Sig. (2-tailed)	,665
		N	12
	VOI-BS_NUK_Group	Correlation Coefficient	,014
		Sig. (2-tailed)	,966
		N	12
	Frontal cortex	Correlation Coefficient	,119
		Sig. (2-tailed)	,713
		N	12
	Orbitofronta cortex	Correlation Coefficient	-,126
		Sig. (2-tailed)	,697
		N	12
	Dorsolateral prefrontal cortex	Correlation Coefficient	-,021
		Sig. (2-tailed)	,948
		N	12
	Anterior cingulate cortex	Correlation Coefficient	-,259
		Sig. (2-tailed)	,417
		N	12
	Insula	Correlation Coefficient	-,028
		Sig. (2-tailed)	,931
		N	12
	Hippocampus	Correlation Coefficient	,350
		Sig. (2-tailed)	,265
		N	12
	Amygdala	Correlation Coefficient	,552
		Sig. (2-tailed)	,063
		N	12

Correlations[a]

			Head of the caudate	Putamen
Spearman's rho	Stress-ACTH-max	Correlation Coefficient	-,245	-,098
		Sig. (2-tailed)	,443	,762
		N	12	12
	Stress-A-AUC	Correlation Coefficient	-,182	-,028
		Sig. (2-tailed)	,572	,931
		N	12	12
	Stress-C-max	Correlation Coefficient	-,315	-,056
		Sig. (2-tailed)	,319	,863
		N	12	12
	Stress-C-AUC	Correlation Coefficient	-,336	-,112
		Sig. (2-tailed)	,286	,729
		N	12	12
	VOI-BS_NUK_Group	Correlation Coefficient	,448	,531
		Sig. (2-tailed)	,145	,075
		N	12	12
	Frontal cortex	Correlation Coefficient	-,259	-,133
		Sig. (2-tailed)	,417	,681
		N	12	12
	Orbitofronta cortex	Correlation Coefficient	,259	,238
		Sig. (2-tailed)	,417	,457
		N	12	12
	Dorsolateral prefrontal cortex	Correlation Coefficient	-,371	-,280
		Sig. (2-tailed)	,236	,379
		N	12	12
	Anterior cingulate cortex	Correlation Coefficient	-,056	-,014
		Sig. (2-tailed)	,863	,966
		N	12	12
	Insula	Correlation Coefficient	-,056	-,021
		Sig. (2-tailed)	,863	,948
		N	12	12
	Hippocampus	Correlation Coefficient	-,049	,042
		Sig. (2-tailed)	,880	,897
		N	12	12
	Amygdala	Correlation Coefficient	,524	,238
		Sig. (2-tailed)	,080	,457
		N	12	12

Correlations[a]

			Thalamus	Hypothalamus
Spearman's rho	Stress-ACTH-max	Correlation Coefficient	,147	,042
		Sig. (2-tailed)	,649	,897
		N	12	12
	Stress-A-AUC	Correlation Coefficient	,322	,154
		Sig. (2-tailed)	,308	,633
		N	12	12
	Stress-C-max	Correlation Coefficient	,287	,168
		Sig. (2-tailed)	,366	,602
		N	12	12
	Stress-C-AUC	Correlation Coefficient	,245	,175
		Sig. (2-tailed)	,443	,587
		N	12	12
	VOI-BS_NUK_Group	Correlation Coefficient	,692*	,867**
		Sig. (2-tailed)	,013	,000
		N	12	12
	Frontal cortex	Correlation Coefficient	,406	,629*
		Sig. (2-tailed)	,191	,028
		N	12	12
	Orbitofronta cortex	Correlation Coefficient	,105	,378
		Sig. (2-tailed)	,746	,226
		N	12	12
	Dorsolateral prefrontal cortex	Correlation Coefficient	,161	,517
		Sig. (2-tailed)	,618	,085
		N	12	12
	Anterior cingulate cortex	Correlation Coefficient	,084	,385
		Sig. (2-tailed)	,795	,217
		N	12	12
	Insula	Correlation Coefficient	,161	,608*
		Sig. (2-tailed)	,618	,036
		N	12	12
	Hippocampus	Correlation Coefficient	,559	,420
		Sig. (2-tailed)	,059	,175
		N	12	12
	Amygdala	Correlation Coefficient	,112	,231
		Sig. (2-tailed)	,729	,471
		N	12	12

Correlations[a]

			Substantia nigra	Midbrain
Spearman's rho	Stress-ACTH-max	Correlation Coefficient	,049	,119
		Sig. (2-tailed)	,880	,713
		N	12	12
	Stress-A-AUC	Correlation Coefficient	,147	,161
		Sig. (2-tailed)	,649	,618
		N	12	12
	Stress-C-max	Correlation Coefficient	,140	-,056
		Sig. (2-tailed)	,665	,863
		N	12	12
	Stress-C-AUC	Correlation Coefficient	,133	,035
		Sig. (2-tailed)	,681	,914
		N	12	12
	VOI-BS_NUK_Group	Correlation Coefficient	,552	,224
		Sig. (2-tailed)	,063	,484
		N	12	12
	Frontal cortex	Correlation Coefficient	,490	,497
		Sig. (2-tailed)	,106	,101
		N	12	12
	Orbitofronta cortex	Correlation Coefficient	,594*	,364
		Sig. (2-tailed)	,042	,245
		N	12	12
	Dorsolateral prefrontal cortex	Correlation Coefficient	,601*	,685*
		Sig. (2-tailed)	,039	,014
		N	12	12
	Anterior cingulate cortex	Correlation Coefficient	,622*	,483
		Sig. (2-tailed)	,031	,112
		N	12	12
	Insula	Correlation Coefficient	,748**	,685*
		Sig. (2-tailed)	,005	,014
		N	12	12
	Hippocampus	Correlation Coefficient	,308	,573
		Sig. (2-tailed)	,331	,051
		N	12	12
	Amygdala	Correlation Coefficient	,266	,643*
		Sig. (2-tailed)	,404	,024
		N	12	12

Correlations[a]

			Pons
Spearman's rho	Stress-ACTH-max	Correlation Coefficient	-,350
		Sig. (2-tailed)	,265
		N	12
	Stress-A-AUC	Correlation Coefficient	-,329
		Sig. (2-tailed)	,297
		N	12
	Stress-C-max	Correlation Coefficient	,196
		Sig. (2-tailed)	,542
		N	12
	Stress-C-AUC	Correlation Coefficient	,322
		Sig. (2-tailed)	,308
		N	12
	VOI-BS_NUK_Group	Correlation Coefficient	,664*
		Sig. (2-tailed)	,018
		N	12
	Frontal cortex	Correlation Coefficient	,476
		Sig. (2-tailed)	,118
		N	12
	Orbitofronta cortex	Correlation Coefficient	,308
		Sig. (2-tailed)	,331
		N	12
	Dorsolateral prefrontal cortex	Correlation Coefficient	,622*
		Sig. (2-tailed)	,031
		N	12
	Anterior cingulate cortex	Correlation Coefficient	,678*
		Sig. (2-tailed)	,015
		N	12
	Insula	Correlation Coefficient	,573
		Sig. (2-tailed)	,051
		N	12
	Hippocampus	Correlation Coefficient	,259
		Sig. (2-tailed)	,417
		N	12
	Amygdala	Correlation Coefficient	,266
		Sig. (2-tailed)	,404
		N	12

Correlations[a]

			Stress-ACTH-max	Stress-A-AUC
	Ncl. accumbens	Correlation Coefficient	-,140	-,287
		Sig. (2-tailed)	,665	,366
		N	12	12
	Head of the caudate	Correlation Coefficient	-,245	-,182
		Sig. (2-tailed)	,443	,572
		N	12	12
	Putamen	Correlation Coefficient	-,098	-,028
		Sig. (2-tailed)	,762	,931
		N	12	12
	Thalamus	Correlation Coefficient	,147	,322
		Sig. (2-tailed)	,649	,308
		N	12	12
	Hypothalamus	Correlation Coefficient	,042	,154
		Sig. (2-tailed)	,897	,633
		N	12	12
	Substantia nigra	Correlation Coefficient	,049	,147
		Sig. (2-tailed)	,880	,649
		N	12	12
	Midbrain	Correlation Coefficient	,119	,161
		Sig. (2-tailed)	,713	,618
		N	12	12
	Pons	Correlation Coefficient	-,350	-,329
		Sig. (2-tailed)	,265	,297
		N	12	12

Correlations[a]

			Stress-C-max	Stress-C-AUC
	Ncl. accumbens	Correlation Coefficient	-,091	,140
		Sig. (2-tailed)	,779	,665
		N	12	12
	Head of the caudate	Correlation Coefficient	-,315	-,336
		Sig. (2-tailed)	,319	,286
		N	12	12
	Putamen	Correlation Coefficient	-,056	-,112
		Sig. (2-tailed)	,863	,729
		N	12	12
	Thalamus	Correlation Coefficient	,287	,245
		Sig. (2-tailed)	,366	,443
		N	12	12
	Hypothalamus	Correlation Coefficient	,168	,175
		Sig. (2-tailed)	,602	,587
		N	12	12
	Substantia nigra	Correlation Coefficient	,140	,133
		Sig. (2-tailed)	,665	,681
		N	12	12
	Midbrain	Correlation Coefficient	-,056	,035
		Sig. (2-tailed)	,863	,914
		N	12	12
	Pons	Correlation Coefficient	,196	,322
		Sig. (2-tailed)	,542	,308
		N	12	12

Correlations[a]

			VOI-BS_NUK_Group	Frontal cortex
	Ncl. accumbens	Correlation Coefficient	,014	,119
		Sig. (2-tailed)	,966	,713
		N	12	12
	Head of the caudate	Correlation Coefficient	,448	-,259
		Sig. (2-tailed)	,145	,417
		N	12	12
	Putamen	Correlation Coefficient	,531	-,133
		Sig. (2-tailed)	,075	,681
		N	12	12
	Thalamus	Correlation Coefficient	,692*	,406
		Sig. (2-tailed)	,013	,191
		N	12	12
	Hypothalamus	Correlation Coefficient	,867**	,629*
		Sig. (2-tailed)	,000	,028
		N	12	12
	Substantia nigra	Correlation Coefficient	,552	,490
		Sig. (2-tailed)	,063	,106
		N	12	12
	Midbrain	Correlation Coefficient	,224	,497
		Sig. (2-tailed)	,484	,101
		N	12	12
	Pons	Correlation Coefficient	,664*	,476
		Sig. (2-tailed)	,018	,118
		N	12	12

Correlations[a]

			Orbitofronta cortex
	Ncl. accumbens	Correlation Coefficient	-,126
		Sig. (2-tailed)	,697
		N	12
	Head of the caudate	Correlation Coefficient	,259
		Sig. (2-tailed)	,417
		N	12
	Putamen	Correlation Coefficient	,238
		Sig. (2-tailed)	,457
		N	12
	Thalamus	Correlation Coefficient	,105
		Sig. (2-tailed)	,746
		N	12
	Hypothalamus	Correlation Coefficient	,378
		Sig. (2-tailed)	,226
		N	12
	Substantia nigra	Correlation Coefficient	,594*
		Sig. (2-tailed)	,042
		N	12
	Midbrain	Correlation Coefficient	,364
		Sig. (2-tailed)	,245
		N	12
	Pons	Correlation Coefficient	,308
		Sig. (2-tailed)	,331
		N	12

Correlations[a]

			Dorsolateral prefrontal cortex
	Ncl. accumbens	Correlation Coefficient	-,021
		Sig. (2-tailed)	,948
		N	12
	Head of the caudate	Correlation Coefficient	-,371
		Sig. (2-tailed)	,236
		N	12
	Putamen	Correlation Coefficient	-,280
		Sig. (2-tailed)	,379
		N	12
	Thalamus	Correlation Coefficient	,161
		Sig. (2-tailed)	,618
		N	12
	Hypothalamus	Correlation Coefficient	,517
		Sig. (2-tailed)	,085
		N	12
	Substantia nigra	Correlation Coefficient	,601*
		Sig. (2-tailed)	,039
		N	12
	Midbrain	Correlation Coefficient	,685*
		Sig. (2-tailed)	,014
		N	12
	Pons	Correlation Coefficient	,622*
		Sig. (2-tailed)	,031
		N	12

Correlations[a]

			Anterior cingulate cortex	Insula
	Ncl. accumbens	Correlation Coefficient	-,259	-,028
		Sig. (2-tailed)	,417	,931
		N	12	12
	Head of the caudate	Correlation Coefficient	-,056	-,056
		Sig. (2-tailed)	,863	,863
		N	12	12
	Putamen	Correlation Coefficient	-,014	-,021
		Sig. (2-tailed)	,966	,948
		N	12	12
	Thalamus	Correlation Coefficient	,084	,161
		Sig. (2-tailed)	,795	,618
		N	12	12
	Hypothalamus	Correlation Coefficient	,385	,608*
		Sig. (2-tailed)	,217	,036
		N	12	12
	Substantia nigra	Correlation Coefficient	,622*	,748**
		Sig. (2-tailed)	,031	,005
		N	12	12
	Midbrain	Correlation Coefficient	,483	,685*
		Sig. (2-tailed)	,112	,014
		N	12	12
	Pons	Correlation Coefficient	,678*	,573
		Sig. (2-tailed)	,015	,051
		N	12	12

Correlations[a]

			Hippocampus	Amygdala
	Ncl. accumbens	Correlation Coefficient	,350	,552
		Sig. (2-tailed)	,265	,063
		N	12	12
	Head of the caudate	Correlation Coefficient	-,049	,524
		Sig. (2-tailed)	,880	,080
		N	12	12
	Putamen	Correlation Coefficient	,042	,238
		Sig. (2-tailed)	,897	,457
		N	12	12
	Thalamus	Correlation Coefficient	,559	,112
		Sig. (2-tailed)	,059	,729
		N	12	12
	Hypothalamus	Correlation Coefficient	,420	,231
		Sig. (2-tailed)	,175	,471
		N	12	12
	Substantia nigra	Correlation Coefficient	,308	,266
		Sig. (2-tailed)	,331	,404
		N	12	12
	Midbrain	Correlation Coefficient	,573	,643*
		Sig. (2-tailed)	,051	,024
		N	12	12
	Pons	Correlation Coefficient	,259	,266
		Sig. (2-tailed)	,417	,404
		N	12	12

Correlations[a]

			Ncl. accumbens
	Ncl. accumbens	Correlation Coefficient	1,000
		Sig. (2-tailed)	.
		N	12
	Head of the caudate	Correlation Coefficient	,399
		Sig. (2-tailed)	,199
		N	12
	Putamen	Correlation Coefficient	,287
		Sig. (2-tailed)	,366
		N	12
	Thalamus	Correlation Coefficient	,182
		Sig. (2-tailed)	,572
		N	12
	Hypothalamus	Correlation Coefficient	,196
		Sig. (2-tailed)	,542
		N	12
	Substantia nigra	Correlation Coefficient	,021
		Sig. (2-tailed)	,948
		N	12
	Midbrain	Correlation Coefficient	,273
		Sig. (2-tailed)	,391
		N	12
	Pons	Correlation Coefficient	,147
		Sig. (2-tailed)	,649
		N	12

Correlations[a]

		Head of the caudate	Putamen
Ncl. accumbens	Correlation Coefficient	,399	,287
	Sig. (2-tailed)	,199	,366
	N	12	12
Head of the caudate	Correlation Coefficient	1,000	,909**
	Sig. (2-tailed)	.	,000
	N	12	12
Putamen	Correlation Coefficient	,909**	1,000
	Sig. (2-tailed)	,000	.
	N	12	12
Thalamus	Correlation Coefficient	,476	,538
	Sig. (2-tailed)	,118	,071
	N	12	12
Hypothalamus	Correlation Coefficient	,343	,455
	Sig. (2-tailed)	,276	,138
	N	12	12
Substantia nigra	Correlation Coefficient	,168	,336
	Sig. (2-tailed)	,602	,286
	N	12	12
Midbrain	Correlation Coefficient	-,042	-,119
	Sig. (2-tailed)	,897	,713
	N	12	12
Pons	Correlation Coefficient	,154	,252
	Sig. (2-tailed)	,633	,430
	N	12	12

Correlations[a]

			Thalamus	Hypothalamus
	Ncl. accumbens	Correlation Coefficient	,182	,196
		Sig. (2-tailed)	,572	,542
		N	12	12
	Head of the caudate	Correlation Coefficient	,476	,343
		Sig. (2-tailed)	,118	,276
		N	12	12
	Putamen	Correlation Coefficient	,538	,455
		Sig. (2-tailed)	,071	,138
		N	12	12
	Thalamus	Correlation Coefficient	1,000	,636*
		Sig. (2-tailed)	.	,026
		N	12	12
	Hypothalamus	Correlation Coefficient	,636*	1,000
		Sig. (2-tailed)	,026	.
		N	12	12
	Substantia nigra	Correlation Coefficient	,126	,650*
		Sig. (2-tailed)	,697	,022
		N	12	12
	Midbrain	Correlation Coefficient	,070	,406
		Sig. (2-tailed)	,829	,191
		N	12	12
	Pons	Correlation Coefficient	,294	,685*
		Sig. (2-tailed)	,354	,014
		N	12	12

Correlations[a]

			Substantia nigra	Midbrain
	Ncl. accumbens	Correlation Coefficient	,021	,273
		Sig. (2-tailed)	,948	,391
		N	12	12
	Head of the caudate	Correlation Coefficient	,168	-,042
		Sig. (2-tailed)	,602	,897
		N	12	12
	Putamen	Correlation Coefficient	,336	-,119
		Sig. (2-tailed)	,286	,713
		N	12	12
	Thalamus	Correlation Coefficient	,126	,070
		Sig. (2-tailed)	,697	,829
		N	12	12
	Hypothalamus	Correlation Coefficient	,650*	,406
		Sig. (2-tailed)	,022	,191
		N	12	12
	Substantia nigra	Correlation Coefficient	1,000	,657*
		Sig. (2-tailed)	.	,020
		N	12	12
	Midbrain	Correlation Coefficient	,657*	1,000
		Sig. (2-tailed)	,020	.
		N	12	12
	Pons	Correlation Coefficient	,524	,308
		Sig. (2-tailed)	,080	,331
		N	12	12

Correlations[a]

			Pons
	Ncl. accumbens	Correlation Coefficient	,147
		Sig. (2-tailed)	,649
		N	12
	Head of the caudate	Correlation Coefficient	,154
		Sig. (2-tailed)	,633
		N	12
	Putamen	Correlation Coefficient	,252
		Sig. (2-tailed)	,430
		N	12
	Thalamus	Correlation Coefficient	,294
		Sig. (2-tailed)	,354
		N	12
	Hypothalamus	Correlation Coefficient	,685*
		Sig. (2-tailed)	,014
		N	12
	Substantia nigra	Correlation Coefficient	,524
		Sig. (2-tailed)	,080
		N	12
	Midbrain	Correlation Coefficient	,308
		Sig. (2-tailed)	,331
		N	12
	Pons	Correlation Coefficient	1,000
		Sig. (2-tailed)	.
		N	12

**. Correlation is significant at the 0.01 level (2-tailed).

*. Correlation is significant at the 0.05 level (2-tailed).

a. Status
(0=NC,1=ADI) = LC

Status
(0=NC,1=ADI) = OB

Correlations[a]

			Stress-ACTH-max	Stress-A-AUC
Spearman's rho	Stress-ACTH-max	Correlation Coefficient	1,000	,987**
		Sig. (2-tailed)	.	,000
		N	28	28
	Stress-A-AUC	Correlation Coefficient	,987**	1,000
		Sig. (2-tailed)	,000	.
		N	28	28
	Stress-C-max	Correlation Coefficient	,442*	,452*
		Sig. (2-tailed)	,019	,016
		N	28	28
	Stress-C-AUC	Correlation Coefficient	,446*	,462*
		Sig. (2-tailed)	,017	,013
		N	28	28
	VOI-BS_NUK_Group	Correlation Coefficient	,389*	,393*
		Sig. (2-tailed)	,041	,039
		N	28	28
	Frontal cortex	Correlation Coefficient	,272	,288
		Sig. (2-tailed)	,161	,137
		N	28	28
	Orbitofronta cortex	Correlation Coefficient	,336	,350
		Sig. (2-tailed)	,081	,068
		N	28	28
	Dorsolateral prefrontal cortex	Correlation Coefficient	,290	,328
		Sig. (2-tailed)	,135	,089
		N	28	28
	Anterior cingulate cortex	Correlation Coefficient	,287	,303
		Sig. (2-tailed)	,139	,117
		N	28	28
	Insula	Correlation Coefficient	,216	,203
		Sig. (2-tailed)	,269	,300
		N	28	28
	Hippocampus	Correlation Coefficient	,035	,048
		Sig. (2-tailed)	,859	,808
		N	28	28
	Amygdala	Correlation Coefficient	,228	,238
		Sig. (2-tailed)	,243	,224
		N	28	28

Correlations[a]

			Stress-C-max	Stress-C-AUC
Spearman's rho	Stress-ACTH-max	Correlation Coefficient	,442*	,446*
		Sig. (2-tailed)	,019	,017
		N	28	28
	Stress-A-AUC	Correlation Coefficient	,452*	,462*
		Sig. (2-tailed)	,016	,013
		N	28	28
	Stress-C-max	Correlation Coefficient	1,000	,991**
		Sig. (2-tailed)	.	,000
		N	28	28
	Stress-C-AUC	Correlation Coefficient	,991**	1,000
		Sig. (2-tailed)	,000	.
		N	28	28
	VOI-BS_NUK_Group	Correlation Coefficient	,151	,089
		Sig. (2-tailed)	,443	,654
		N	28	28
	Frontal cortex	Correlation Coefficient	,021	-,004
		Sig. (2-tailed)	,916	,985
		N	28	28
	Orbitofronta cortex	Correlation Coefficient	,047	,010
		Sig. (2-tailed)	,812	,958
		N	28	28
	Dorsolateral prefrontal cortex	Correlation Coefficient	-,070	-,111
		Sig. (2-tailed)	,723	,575
		N	28	28
	Anterior cingulate cortex	Correlation Coefficient	,113	,081
		Sig. (2-tailed)	,566	,682
		N	28	28
	Insula	Correlation Coefficient	,114	,062
		Sig. (2-tailed)	,562	,752
		N	28	28
	Hippocampus	Correlation Coefficient	-,044	-,074
		Sig. (2-tailed)	,825	,707
		N	28	28
	Amygdala	Correlation Coefficient	-,034	-,081
		Sig. (2-tailed)	,862	,682
		N	28	28

Correlations[a]

			VOI-BS_NUK_Group	Frontal cortex
Spearman's rho	Stress-ACTH-max	Correlation Coefficient	,389*	,272
		Sig. (2-tailed)	,041	,161
		N	28	28
	Stress-A-AUC	Correlation Coefficient	,393*	,288
		Sig. (2-tailed)	,039	,137
		N	28	28
	Stress-C-max	Correlation Coefficient	,151	,021
		Sig. (2-tailed)	,443	,916
		N	28	28
	Stress-C-AUC	Correlation Coefficient	,089	-,004
		Sig. (2-tailed)	,654	,985
		N	28	28
	VOI-BS_NUK_Group	Correlation Coefficient	1,000	,724**
		Sig. (2-tailed)	.	,000
		N	28	28
	Frontal cortex	Correlation Coefficient	,724**	1,000
		Sig. (2-tailed)	,000	.
		N	28	28
	Orbitofronta cortex	Correlation Coefficient	,721**	,625**
		Sig. (2-tailed)	,000	,000
		N	28	28
	Dorsolateral prefrontal cortex	Correlation Coefficient	,829**	,837**
		Sig. (2-tailed)	,000	,000
		N	28	28
	Anterior cingulate cortex	Correlation Coefficient	,874**	,738**
		Sig. (2-tailed)	,000	,000
		N	28	28
	Insula	Correlation Coefficient	,886**	,736**
		Sig. (2-tailed)	,000	,000
		N	28	28
	Hippocampus	Correlation Coefficient	,738**	,668**
		Sig. (2-tailed)	,000	,000
		N	28	28
	Amygdala	Correlation Coefficient	,847**	,543**
		Sig. (2-tailed)	,000	,003
		N	28	28

Correlations[a]

			Orbitofronta cortex
Spearman's rho	Stress-ACTH-max	Correlation Coefficient	,336
		Sig. (2-tailed)	,081
		N	28
	Stress-A-AUC	Correlation Coefficient	,350
		Sig. (2-tailed)	,068
		N	28
	Stress-C-max	Correlation Coefficient	,047
		Sig. (2-tailed)	,812
		N	28
	Stress-C-AUC	Correlation Coefficient	,010
		Sig. (2-tailed)	,958
		N	28
	VOI-BS_NUK_Group	Correlation Coefficient	,721**
		Sig. (2-tailed)	,000
		N	28
	Frontal cortex	Correlation Coefficient	,625**
		Sig. (2-tailed)	,000
		N	28
	Orbitofronta cortex	Correlation Coefficient	1,000
		Sig. (2-tailed)	.
		N	28
	Dorsolateral prefrontal cortex	Correlation Coefficient	,646**
		Sig. (2-tailed)	,000
		N	28
	Anterior cingulate cortex	Correlation Coefficient	,581**
		Sig. (2-tailed)	,001
		N	28
	Insula	Correlation Coefficient	,663**
		Sig. (2-tailed)	,000
		N	28
	Hippocampus	Correlation Coefficient	,508**
		Sig. (2-tailed)	,006
		N	28
	Amygdala	Correlation Coefficient	,587**
		Sig. (2-tailed)	,001
		N	28

Correlations[a]

			Dorsolateral prefrontal cortex
Spearman's rho	Stress-ACTH-max	Correlation Coefficient	,290
		Sig. (2-tailed)	,135
		N	28
	Stress-A-AUC	Correlation Coefficient	,328
		Sig. (2-tailed)	,089
		N	28
	Stress-C-max	Correlation Coefficient	-,070
		Sig. (2-tailed)	,723
		N	28
	Stress-C-AUC	Correlation Coefficient	-,111
		Sig. (2-tailed)	,575
		N	28
	VOI-BS_NUK_Group	Correlation Coefficient	,829**
		Sig. (2-tailed)	,000
		N	28
	Frontal cortex	Correlation Coefficient	,837**
		Sig. (2-tailed)	,000
		N	28
	Orbitofronta cortex	Correlation Coefficient	,646**
		Sig. (2-tailed)	,000
		N	28
	Dorsolateral prefrontal cortex	Correlation Coefficient	1,000
		Sig. (2-tailed)	.
		N	28
	Anterior cingulate cortex	Correlation Coefficient	,828**
		Sig. (2-tailed)	,000
		N	28
	Insula	Correlation Coefficient	,765**
		Sig. (2-tailed)	,000
		N	28
	Hippocampus	Correlation Coefficient	,628**
		Sig. (2-tailed)	,000
		N	28
	Amygdala	Correlation Coefficient	,686**
		Sig. (2-tailed)	,000
		N	28

Correlations[a]

			Anterior cingulate cortex	Insula
Spearman's rho	Stress-ACTH-max	Correlation Coefficient	,287	,216
		Sig. (2-tailed)	,139	,269
		N	28	28
	Stress-A-AUC	Correlation Coefficient	,303	,203
		Sig. (2-tailed)	,117	,300
		N	28	28
	Stress-C-max	Correlation Coefficient	,113	,114
		Sig. (2-tailed)	,566	,562
		N	28	28
	Stress-C-AUC	Correlation Coefficient	,081	,062
		Sig. (2-tailed)	,682	,752
		N	28	28
	VOI-BS_NUK_Group	Correlation Coefficient	,874**	,886**
		Sig. (2-tailed)	,000	,000
		N	28	28
	Frontal cortex	Correlation Coefficient	,738**	,736**
		Sig. (2-tailed)	,000	,000
		N	28	28
	Orbitofronta cortex	Correlation Coefficient	,581**	,663**
		Sig. (2-tailed)	,001	,000
		N	28	28
	Dorsolateral prefrontal cortex	Correlation Coefficient	,828**	,765**
		Sig. (2-tailed)	,000	,000
		N	28	28
	Anterior cingulate cortex	Correlation Coefficient	1,000	,859**
		Sig. (2-tailed)	.	,000
		N	28	28
	Insula	Correlation Coefficient	,859**	1,000
		Sig. (2-tailed)	,000	.
		N	28	28
	Hippocampus	Correlation Coefficient	,651**	,759**
		Sig. (2-tailed)	,000	,000
		N	28	28
	Amygdala	Correlation Coefficient	,830**	,828**
		Sig. (2-tailed)	,000	,000
		N	28	28

Correlations[a]

			Hippocampus	Amygdala
Spearman's rho	Stress-ACTH-max	Correlation Coefficient	,035	,228
		Sig. (2-tailed)	,859	,243
		N	28	28
	Stress-A-AUC	Correlation Coefficient	,048	,238
		Sig. (2-tailed)	,808	,224
		N	28	28
	Stress-C-max	Correlation Coefficient	-,044	-,034
		Sig. (2-tailed)	,825	,862
		N	28	28
	Stress-C-AUC	Correlation Coefficient	-,074	-,081
		Sig. (2-tailed)	,707	,682
		N	28	28
	VOI-BS_NUK_Group	Correlation Coefficient	,738**	,847**
		Sig. (2-tailed)	,000	,000
		N	28	28
	Frontal cortex	Correlation Coefficient	,668**	,543**
		Sig. (2-tailed)	,000	,003
		N	28	28
	Orbitofronta cortex	Correlation Coefficient	,508**	,587**
		Sig. (2-tailed)	,006	,001
		N	28	28
	Dorsolateral prefrontal cortex	Correlation Coefficient	,628**	,686**
		Sig. (2-tailed)	,000	,000
		N	28	28
	Anterior cingulate cortex	Correlation Coefficient	,651**	,830**
		Sig. (2-tailed)	,000	,000
		N	28	28
	Insula	Correlation Coefficient	,759**	,828**
		Sig. (2-tailed)	,000	,000
		N	28	28
	Hippocampus	Correlation Coefficient	1,000	,619**
		Sig. (2-tailed)	.	,000
		N	28	28
	Amygdala	Correlation Coefficient	,619**	1,000
		Sig. (2-tailed)	,000	.
		N	28	28

Correlations[a]

			Ncl. accumbens
Spearman's rho	Stress-ACTH-max	Correlation Coefficient	,322
		Sig. (2-tailed)	,094
		N	28
	Stress-A-AUC	Correlation Coefficient	,291
		Sig. (2-tailed)	,134
		N	28
	Stress-C-max	Correlation Coefficient	,107
		Sig. (2-tailed)	,589
		N	28
	Stress-C-AUC	Correlation Coefficient	,048
		Sig. (2-tailed)	,808
		N	28
	VOI-BS_NUK_Group	Correlation Coefficient	,759**
		Sig. (2-tailed)	,000
		N	28
	Frontal cortex	Correlation Coefficient	,459*
		Sig. (2-tailed)	,014
		N	28
	Orbitofronta cortex	Correlation Coefficient	,675**
		Sig. (2-tailed)	,000
		N	28
	Dorsolateral prefrontal cortex	Correlation Coefficient	,524**
		Sig. (2-tailed)	,004
		N	28
	Anterior cingulate cortex	Correlation Coefficient	,666**
		Sig. (2-tailed)	,000
		N	28
	Insula	Correlation Coefficient	,744**
		Sig. (2-tailed)	,000
		N	28
	Hippocampus	Correlation Coefficient	,372
		Sig. (2-tailed)	,051
		N	28
	Amygdala	Correlation Coefficient	,756**
		Sig. (2-tailed)	,000
		N	28

Correlations[a]

			Head of the caudate	Putamen
Spearman's rho	Stress-ACTH-max	Correlation Coefficient	,575**	,279
		Sig. (2-tailed)	,001	,151
		N	28	28
	Stress-A-AUC	Correlation Coefficient	,540**	,236
		Sig. (2-tailed)	,003	,227
		N	28	28
	Stress-C-max	Correlation Coefficient	,213	,049
		Sig. (2-tailed)	,275	,806
		N	28	28
	Stress-C-AUC	Correlation Coefficient	,149	-,019
		Sig. (2-tailed)	,450	,925
		N	28	28
	VOI-BS_NUK_Group	Correlation Coefficient	,856**	,797**
		Sig. (2-tailed)	,000	,000
		N	28	28
	Frontal cortex	Correlation Coefficient	,472*	,340
		Sig. (2-tailed)	,011	,076
		N	28	28
	Orbitofronta cortex	Correlation Coefficient	,643**	,577**
		Sig. (2-tailed)	,000	,001
		N	28	28
	Dorsolateral prefrontal cortex	Correlation Coefficient	,592**	,523**
		Sig. (2-tailed)	,001	,004
		N	28	28
	Anterior cingulate cortex	Correlation Coefficient	,687**	,597**
		Sig. (2-tailed)	,000	,001
		N	28	28
	Insula	Correlation Coefficient	,738**	,690**
		Sig. (2-tailed)	,000	,000
		N	28	28
	Hippocampus	Correlation Coefficient	,525**	,530**
		Sig. (2-tailed)	,004	,004
		N	28	28
	Amygdala	Correlation Coefficient	,744**	,668**
		Sig. (2-tailed)	,000	,000
		N	28	28

Correlations[a]

			Thalamus	Hypothalamus
Spearman's rho	Stress-ACTH-max	Correlation Coefficient	,038	,152
		Sig. (2-tailed)	,849	,439
		N	28	28
	Stress-A-AUC	Correlation Coefficient	,027	,124
		Sig. (2-tailed)	,890	,531
		N	28	28
	Stress-C-max	Correlation Coefficient	-,111	-,056
		Sig. (2-tailed)	,575	,778
		N	28	28
	Stress-C-AUC	Correlation Coefficient	-,177	-,136
		Sig. (2-tailed)	,368	,491
		N	28	28
	VOI-BS_NUK_Group	Correlation Coefficient	,713**	,797**
		Sig. (2-tailed)	,000	,000
		N	28	28
	Frontal cortex	Correlation Coefficient	,356	,395*
		Sig. (2-tailed)	,063	,038
		N	28	28
	Orbitofronta cortex	Correlation Coefficient	,459*	,538**
		Sig. (2-tailed)	,014	,003
		N	28	28
	Dorsolateral prefrontal cortex	Correlation Coefficient	,521**	,640**
		Sig. (2-tailed)	,004	,000
		N	28	28
	Anterior cingulate cortex	Correlation Coefficient	,540**	,680**
		Sig. (2-tailed)	,003	,000
		N	28	28
	Insula	Correlation Coefficient	,697**	,698**
		Sig. (2-tailed)	,000	,000
		N	28	28
	Hippocampus	Correlation Coefficient	,539**	,448*
		Sig. (2-tailed)	,003	,017
		N	28	28
	Amygdala	Correlation Coefficient	,687**	,733**
		Sig. (2-tailed)	,000	,000
		N	28	28

Correlations[a]

			Substantia nigra	Midbrain
Spearman's rho	Stress-ACTH-max	Correlation Coefficient	,198	,244
		Sig. (2-tailed)	,312	,212
		N	28	28
	Stress-A-AUC	Correlation Coefficient	,186	,217
		Sig. (2-tailed)	,343	,268
		N	28	28
	Stress-C-max	Correlation Coefficient	-,143	-,131
		Sig. (2-tailed)	,467	,505
		N	28	28
	Stress-C-AUC	Correlation Coefficient	-,218	-,217
		Sig. (2-tailed)	,264	,268
		N	28	28
	VOI-BS_NUK_Group	Correlation Coefficient	,741**	,785**
		Sig. (2-tailed)	,000	,000
		N	28	28
	Frontal cortex	Correlation Coefficient	,446*	,475*
		Sig. (2-tailed)	,017	,011
		N	28	28
	Orbitofronta cortex	Correlation Coefficient	,591**	,611**
		Sig. (2-tailed)	,001	,001
		N	28	28
	Dorsolateral prefrontal cortex	Correlation Coefficient	,686**	,699**
		Sig. (2-tailed)	,000	,000
		N	28	28
	Anterior cingulate cortex	Correlation Coefficient	,637**	,611**
		Sig. (2-tailed)	,000	,001
		N	28	28
	Insula	Correlation Coefficient	,650**	,664**
		Sig. (2-tailed)	,000	,000
		N	28	28
	Hippocampus	Correlation Coefficient	,488**	,533**
		Sig. (2-tailed)	,008	,004
		N	28	28
	Amygdala	Correlation Coefficient	,684**	,663**
		Sig. (2-tailed)	,000	,000
		N	28	28

Correlations[a]

			Pons
Spearman's rho	Stress-ACTH-max	Correlation Coefficient	,053
		Sig. (2-tailed)	,791
		N	28
	Stress-A-AUC	Correlation Coefficient	,044
		Sig. (2-tailed)	,825
		N	28
	Stress-C-max	Correlation Coefficient	-,089
		Sig. (2-tailed)	,652
		N	28
	Stress-C-AUC	Correlation Coefficient	-,176
		Sig. (2-tailed)	,370
		N	28
	VOI-BS_NUK_Group	Correlation Coefficient	,737**
		Sig. (2-tailed)	,000
		N	28
	Frontal cortex	Correlation Coefficient	,390*
		Sig. (2-tailed)	,040
		N	28
	Orbitofronta cortex	Correlation Coefficient	,518**
		Sig. (2-tailed)	,005
		N	28
	Dorsolateral prefrontal cortex	Correlation Coefficient	,649**
		Sig. (2-tailed)	,000
		N	28
	Anterior cingulate cortex	Correlation Coefficient	,609**
		Sig. (2-tailed)	,001
		N	28
	Insula	Correlation Coefficient	,739**
		Sig. (2-tailed)	,000
		N	28
	Hippocampus	Correlation Coefficient	,510**
		Sig. (2-tailed)	,006
		N	28
	Amygdala	Correlation Coefficient	,701**
		Sig. (2-tailed)	,000
		N	28

Correlations[a]

			Stress-ACTH-max	Stress-A-AUC
	Ncl. accumbens	Correlation Coefficient	,322	,291
		Sig. (2-tailed)	,094	,134
		N	28	28
	Head of the caudate	Correlation Coefficient	,575**	,540**
		Sig. (2-tailed)	,001	,003
		N	28	28
	Putamen	Correlation Coefficient	,279	,236
		Sig. (2-tailed)	,151	,227
		N	28	28
	Thalamus	Correlation Coefficient	,038	,027
		Sig. (2-tailed)	,849	,890
		N	28	28
	Hypothalamus	Correlation Coefficient	,152	,124
		Sig. (2-tailed)	,439	,531
		N	28	28
	Substantia nigra	Correlation Coefficient	,198	,186
		Sig. (2-tailed)	,312	,343
		N	28	28
	Midbrain	Correlation Coefficient	,244	,217
		Sig. (2-tailed)	,212	,268
		N	28	28
	Pons	Correlation Coefficient	,053	,044
		Sig. (2-tailed)	,791	,825
		N	28	28

Correlations[a]

			Stress-C-max	Stress-C-AUC
	Ncl. accumbens	Correlation Coefficient	,107	,048
		Sig. (2-tailed)	,589	,808
		N	28	28
	Head of the caudate	Correlation Coefficient	,213	,149
		Sig. (2-tailed)	,275	,450
		N	28	28
	Putamen	Correlation Coefficient	,049	-,019
		Sig. (2-tailed)	,806	,925
		N	28	28
	Thalamus	Correlation Coefficient	-,111	-,177
		Sig. (2-tailed)	,575	,368
		N	28	28
	Hypothalamus	Correlation Coefficient	-,056	-,136
		Sig. (2-tailed)	,778	,491
		N	28	28
	Substantia nigra	Correlation Coefficient	-,143	-,218
		Sig. (2-tailed)	,467	,264
		N	28	28
	Midbrain	Correlation Coefficient	-,131	-,217
		Sig. (2-tailed)	,505	,268
		N	28	28
	Pons	Correlation Coefficient	-,089	-,176
		Sig. (2-tailed)	,652	,370
		N	28	28

Correlations[a]

			VOI-BS_NUK_Group	Frontal cortex
	Ncl. accumbens	Correlation Coefficient	,759**	,459*
		Sig. (2-tailed)	,000	,014
		N	28	28
	Head of the caudate	Correlation Coefficient	,856**	,472*
		Sig. (2-tailed)	,000	,011
		N	28	28
	Putamen	Correlation Coefficient	,797**	,340
		Sig. (2-tailed)	,000	,076
		N	28	28
	Thalamus	Correlation Coefficient	,713**	,356
		Sig. (2-tailed)	,000	,063
		N	28	28
	Hypothalamus	Correlation Coefficient	,797**	,395*
		Sig. (2-tailed)	,000	,038
		N	28	28
	Substantia nigra	Correlation Coefficient	,741**	,446*
		Sig. (2-tailed)	,000	,017
		N	28	28
	Midbrain	Correlation Coefficient	,785**	,475*
		Sig. (2-tailed)	,000	,011
		N	28	28
	Pons	Correlation Coefficient	,737**	,390*
		Sig. (2-tailed)	,000	,040
		N	28	28

Correlations[a]

			Orbitofronta cortex
	Ncl. accumbens	Correlation Coefficient	,675**
		Sig. (2-tailed)	,000
		N	28
	Head of the caudate	Correlation Coefficient	,643**
		Sig. (2-tailed)	,000
		N	28
	Putamen	Correlation Coefficient	,577**
		Sig. (2-tailed)	,001
		N	28
	Thalamus	Correlation Coefficient	,459*
		Sig. (2-tailed)	,014
		N	28
	Hypothalamus	Correlation Coefficient	,538**
		Sig. (2-tailed)	,003
		N	28
	Substantia nigra	Correlation Coefficient	,591**
		Sig. (2-tailed)	,001
		N	28
	Midbrain	Correlation Coefficient	,611**
		Sig. (2-tailed)	,001
		N	28
	Pons	Correlation Coefficient	,518**
		Sig. (2-tailed)	,005
		N	28

Correlations[a]

			Dorsolateral prefrontal cortex
	Ncl. accumbens	Correlation Coefficient	,524**
		Sig. (2-tailed)	,004
		N	28
	Head of the caudate	Correlation Coefficient	,592**
		Sig. (2-tailed)	,001
		N	28
	Putamen	Correlation Coefficient	,523**
		Sig. (2-tailed)	,004
		N	28
	Thalamus	Correlation Coefficient	,521**
		Sig. (2-tailed)	,004
		N	28
	Hypothalamus	Correlation Coefficient	,640**
		Sig. (2-tailed)	,000
		N	28
	Substantia nigra	Correlation Coefficient	,686**
		Sig. (2-tailed)	,000
		N	28
	Midbrain	Correlation Coefficient	,699**
		Sig. (2-tailed)	,000
		N	28
	Pons	Correlation Coefficient	,649**
		Sig. (2-tailed)	,000
		N	28

Correlations[a]

			Anterior cingulate cortex	Insula
	Ncl. accumbens	Correlation Coefficient	,666**	,744**
		Sig. (2-tailed)	,000	,000
		N	28	28
	Head of the caudate	Correlation Coefficient	,687**	,738**
		Sig. (2-tailed)	,000	,000
		N	28	28
	Putamen	Correlation Coefficient	,597**	,690**
		Sig. (2-tailed)	,001	,000
		N	28	28
	Thalamus	Correlation Coefficient	,540**	,697**
		Sig. (2-tailed)	,003	,000
		N	28	28
	Hypothalamus	Correlation Coefficient	,680**	,698**
		Sig. (2-tailed)	,000	,000
		N	28	28
	Substantia nigra	Correlation Coefficient	,637**	,650**
		Sig. (2-tailed)	,000	,000
		N	28	28
	Midbrain	Correlation Coefficient	,611**	,664**
		Sig. (2-tailed)	,001	,000
		N	28	28
	Pons	Correlation Coefficient	,609**	,739**
		Sig. (2-tailed)	,001	,000
		N	28	28

Correlations[a]

			Hippocampus	Amygdala
	Ncl. accumbens	Correlation Coefficient	,372	,756**
		Sig. (2-tailed)	,051	,000
		N	28	28
	Head of the caudate	Correlation Coefficient	,525**	,744**
		Sig. (2-tailed)	,004	,000
		N	28	28
	Putamen	Correlation Coefficient	,530**	,668**
		Sig. (2-tailed)	,004	,000
		N	28	28
	Thalamus	Correlation Coefficient	,539**	,687**
		Sig. (2-tailed)	,003	,000
		N	28	28
	Hypothalamus	Correlation Coefficient	,448*	,733**
		Sig. (2-tailed)	,017	,000
		N	28	28
	Substantia nigra	Correlation Coefficient	,488**	,684**
		Sig. (2-tailed)	,008	,000
		N	28	28
	Midbrain	Correlation Coefficient	,533**	,663**
		Sig. (2-tailed)	,004	,000
		N	28	28
	Pons	Correlation Coefficient	,510**	,701**
		Sig. (2-tailed)	,006	,000
		N	28	28

Correlations[a]

			Ncl. accumbens
	Ncl. accumbens	Correlation Coefficient	1,000
		Sig. (2-tailed)	.
		N	28
	Head of the caudate	Correlation Coefficient	,807**
		Sig. (2-tailed)	,000
		N	28
	Putamen	Correlation Coefficient	,804**
		Sig. (2-tailed)	,000
		N	28
	Thalamus	Correlation Coefficient	,584**
		Sig. (2-tailed)	,001
		N	28
	Hypothalamus	Correlation Coefficient	,727**
		Sig. (2-tailed)	,000
		N	28
	Substantia nigra	Correlation Coefficient	,680**
		Sig. (2-tailed)	,000
		N	28
	Midbrain	Correlation Coefficient	,684**
		Sig. (2-tailed)	,000
		N	28
	Pons	Correlation Coefficient	,634**
		Sig. (2-tailed)	,000
		N	28

Correlations[a]

			Head of the caudate	Putamen
	Ncl. accumbens	Correlation Coefficient	,807**	,804**
		Sig. (2-tailed)	,000	,000
		N	28	28
	Head of the caudate	Correlation Coefficient	1,000	,836**
		Sig. (2-tailed)	.	,000
		N	28	28
	Putamen	Correlation Coefficient	,836**	1,000
		Sig. (2-tailed)	,000	.
		N	28	28
	Thalamus	Correlation Coefficient	,633**	,707**
		Sig. (2-tailed)	,000	,000
		N	28	28
	Hypothalamus	Correlation Coefficient	,727**	,848**
		Sig. (2-tailed)	,000	,000
		N	28	28
	Substantia nigra	Correlation Coefficient	,744**	,707**
		Sig. (2-tailed)	,000	,000
		N	28	28
	Midbrain	Correlation Coefficient	,779**	,788**
		Sig. (2-tailed)	,000	,000
		N	28	28
	Pons	Correlation Coefficient	,664**	,696**
		Sig. (2-tailed)	,000	,000
		N	28	28

Correlations[a]

			Thalamus	Hypothalamus
	Ncl. accumbens	Correlation Coefficient	,584**	,727**
		Sig. (2-tailed)	,001	,000
		N	28	28
	Head of the caudate	Correlation Coefficient	,633**	,727**
		Sig. (2-tailed)	,000	,000
		N	28	28
	Putamen	Correlation Coefficient	,707**	,848**
		Sig. (2-tailed)	,000	,000
		N	28	28
	Thalamus	Correlation Coefficient	1,000	,729**
		Sig. (2-tailed)	.	,000
		N	28	28
	Hypothalamus	Correlation Coefficient	,729**	1,000
		Sig. (2-tailed)	,000	.
		N	28	28
	Substantia nigra	Correlation Coefficient	,621**	,806**
		Sig. (2-tailed)	,000	,000
		N	28	28
	Midbrain	Correlation Coefficient	,643**	,841**
		Sig. (2-tailed)	,000	,000
		N	28	28
	Pons	Correlation Coefficient	,786**	,788**
		Sig. (2-tailed)	,000	,000
		N	28	28

Correlations[a]

			Substantia nigra	Midbrain
	Ncl. accumbens	Correlation Coefficient	,680**	,684**
		Sig. (2-tailed)	,000	,000
		N	28	28
	Head of the caudate	Correlation Coefficient	,744**	,779**
		Sig. (2-tailed)	,000	,000
		N	28	28
	Putamen	Correlation Coefficient	,707**	,788**
		Sig. (2-tailed)	,000	,000
		N	28	28
	Thalamus	Correlation Coefficient	,621**	,643**
		Sig. (2-tailed)	,000	,000
		N	28	28
	Hypothalamus	Correlation Coefficient	,806**	,841**
		Sig. (2-tailed)	,000	,000
		N	28	28
	Substantia nigra	Correlation Coefficient	1,000	,935**
		Sig. (2-tailed)	.	,000
		N	28	28
	Midbrain	Correlation Coefficient	,935**	1,000
		Sig. (2-tailed)	,000	.
		N	28	28
	Pons	Correlation Coefficient	,856**	,832**
		Sig. (2-tailed)	,000	,000
		N	28	28

Correlations[a]

			Pons
	Ncl. accumbens	Correlation Coefficient	,634**
		Sig. (2-tailed)	,000
		N	28
	Head of the caudate	Correlation Coefficient	,664**
		Sig. (2-tailed)	,000
		N	28
	Putamen	Correlation Coefficient	,696**
		Sig. (2-tailed)	,000
		N	28
	Thalamus	Correlation Coefficient	,786**
		Sig. (2-tailed)	,000
		N	28
	Hypothalamus	Correlation Coefficient	,788**
		Sig. (2-tailed)	,000
		N	28
	Substantia nigra	Correlation Coefficient	,856**
		Sig. (2-tailed)	,000
		N	28
	Midbrain	Correlation Coefficient	,832**
		Sig. (2-tailed)	,000
		N	28
	Pons	Correlation Coefficient	1,000
		Sig. (2-tailed)	.
		N	28

**. Correlation is significant at the 0.01 level (2-tailed).

*. Correlation is significant at the 0.05 level (2-tailed).

a. Status (0=NC,1=ADI) = OB

```
NONPAR CORR
  /VARIABLES=ACTH_MAX ACTH_AUC CRT_MAX CRT_AUC BDI SCL_90_tANX BAS_Drive BA
S_FUN BAS_Reward BIS
  /PRINT=SPEARMAN TWOTAIL NOSIG
  /MISSING=PAIRWISE.
```

Nonparametric Correlations

Notes

Output Created		21-SEP-2022 00:01:23
Comments		
Input	Data	C:\Users\Christian Schinke\OneDrive - Charité - Universitätsmedizin Berlin\Promotion und Projekte Leipzig\02_DASB vs. HPA\06_Open_Data\01_Data_public.sav
	Active Dataset	DataSet1
	Filter	NOT INCLUSION_EXCLUSION =9 (FILTER)
	Weight	<none>
	Split File	Status (0=NC,1=ADI)
	N of Rows in Working Data File	40
Missing Value Handling	Definition of Missing	User-defined missing values are treated as missing.
	Cases Used	Statistics for each pair of variables are based on all the cases with valid data for that pair.
Syntax		NONPAR CORR /VARIABLES=ACTH_MAX ACTH_AUC CRT_MAX CRT_AUC BDI SCL_90_tANX BAS_Drive BAS_FUN BAS_Reward BIS /PRINT=SPEARMAN TWOTAIL NOSIG...
Resources	Processor Time	00:00:00,02
	Elapsed Time	00:00:00,02
	Number of Cases Allowed	241979 cases[a]

a. Based on availability of workspace memory

Status
(0=NC,1=ADI) = LC

Correlations[a]

			Stress-ACTH-max	Stress-A-AUC
Spearman's rho	Stress-ACTH-max	Correlation Coefficient	1,000	,944**
		Sig. (2-tailed)	.	,000
		N	12	12
	Stress-A-AUC	Correlation Coefficient	,944**	1,000
		Sig. (2-tailed)	,000	.
		N	12	12
	Stress-C-max	Correlation Coefficient	,566	,524
		Sig. (2-tailed)	,055	,080
		N	12	12
	Stress-C-AUC	Correlation Coefficient	,413	,336
		Sig. (2-tailed)	,183	,286
		N	12	12
	Beck Depression Inventory	Correlation Coefficient	-,188	-,025
		Sig. (2-tailed)	,559	,938
		N	12	12
	SCL_90_tANX	Correlation Coefficient	,286	,311
		Sig. (2-tailed)	,368	,325
		N	12	12
	BAS_Drive	Correlation Coefficient	,171	,160
		Sig. (2-tailed)	,594	,619
		N	12	12
	BAS_FUN	Correlation Coefficient	,697*	,568
		Sig. (2-tailed)	,012	,054
		N	12	12
	BAS_Reward	Correlation Coefficient	,643*	,518
		Sig. (2-tailed)	,024	,085
		N	12	12
	BIS	Correlation Coefficient	,396	,266
		Sig. (2-tailed)	,202	,404
		N	12	12

Correlations[a]

			Stress-C-max	Stress-C-AUC
Spearman's rho	Stress-ACTH-max	Correlation Coefficient	,566	,413
		Sig. (2-tailed)	,055	,183
		N	12	12
	Stress-A-AUC	Correlation Coefficient	,524	,336
		Sig. (2-tailed)	,080	,286
		N	12	12
	Stress-C-max	Correlation Coefficient	1,000	,916**
		Sig. (2-tailed)	.	,000
		N	12	12
	Stress-C-AUC	Correlation Coefficient	,916**	1,000
		Sig. (2-tailed)	,000	.
		N	12	12
	Beck Depression Inventory	Correlation Coefficient	,063	,125
		Sig. (2-tailed)	,847	,698
		N	12	12
	SCL_90_tANX	Correlation Coefficient	,604*	,579*
		Sig. (2-tailed)	,037	,049
		N	12	12
	BAS_Drive	Correlation Coefficient	,076	-,034
		Sig. (2-tailed)	,814	,916
		N	12	12
	BAS_FUN	Correlation Coefficient	,478	,359
		Sig. (2-tailed)	,116	,252
		N	12	12
	BAS_Reward	Correlation Coefficient	,232	,043
		Sig. (2-tailed)	,468	,895
		N	12	12
	BIS	Correlation Coefficient	,280	,149
		Sig. (2-tailed)	,379	,645
		N	12	12

Correlations[a]

			Beck Depression Inventory
Spearman's rho	Stress-ACTH-max	Correlation Coefficient	-,188
		Sig. (2-tailed)	,559
		N	12
	Stress-A-AUC	Correlation Coefficient	-,025
		Sig. (2-tailed)	,938
		N	12
	Stress-C-max	Correlation Coefficient	,063
		Sig. (2-tailed)	,847
		N	12
	Stress-C-AUC	Correlation Coefficient	,125
		Sig. (2-tailed)	,698
		N	12
	Beck Depression Inventory	Correlation Coefficient	1,000
		Sig. (2-tailed)	.
		N	12
	SCL_90_tANX	Correlation Coefficient	,149
		Sig. (2-tailed)	,644
		N	12
	BAS_Drive	Correlation Coefficient	-,170
		Sig. (2-tailed)	,597
		N	12
	BAS_FUN	Correlation Coefficient	-,096
		Sig. (2-tailed)	,766
		N	12
	BAS_Reward	Correlation Coefficient	-,275
		Sig. (2-tailed)	,387
		N	12
	BIS	Correlation Coefficient	,101
		Sig. (2-tailed)	,754
		N	12

Correlations[a]

			SCL_90_tANX	BAS_Drive
Spearman's rho	Stress-ACTH-max	Correlation Coefficient	,286	,171
		Sig. (2-tailed)	,368	,594
		N	12	12
	Stress-A-AUC	Correlation Coefficient	,311	,160
		Sig. (2-tailed)	,325	,619
		N	12	12
	Stress-C-max	Correlation Coefficient	,604*	,076
		Sig. (2-tailed)	,037	,814
		N	12	12
	Stress-C-AUC	Correlation Coefficient	,579*	-,034
		Sig. (2-tailed)	,049	,916
		N	12	12
	Beck Depression Inventory	Correlation Coefficient	,149	-,170
		Sig. (2-tailed)	,644	,597
		N	12	12
	SCL_90_tANX	Correlation Coefficient	1,000	-,280
		Sig. (2-tailed)	.	,378
		N	12	12
	BAS_Drive	Correlation Coefficient	-,280	1,000
		Sig. (2-tailed)	,378	.
		N	12	12
	BAS_FUN	Correlation Coefficient	-,028	,481
		Sig. (2-tailed)	,931	,113
		N	12	12
	BAS_Reward	Correlation Coefficient	,100	,261
		Sig. (2-tailed)	,758	,413
		N	12	12
	BIS	Correlation Coefficient	,095	,083
		Sig. (2-tailed)	,768	,798
		N	12	12

Correlations[a]

			BAS_FUN	BAS_Reward
Spearman's rho	Stress-ACTH-max	Correlation Coefficient	,697*	,643*
		Sig. (2-tailed)	,012	,024
		N	12	12
	Stress-A-AUC	Correlation Coefficient	,568	,518
		Sig. (2-tailed)	,054	,085
		N	12	12
	Stress-C-max	Correlation Coefficient	,478	,232
		Sig. (2-tailed)	,116	,468
		N	12	12
	Stress-C-AUC	Correlation Coefficient	,359	,043
		Sig. (2-tailed)	,252	,895
		N	12	12
	Beck Depression Inventory	Correlation Coefficient	-,096	-,275
		Sig. (2-tailed)	,766	,387
		N	12	12
	SCL_90_tANX	Correlation Coefficient	-,028	,100
		Sig. (2-tailed)	,931	,758
		N	12	12
	BAS_Drive	Correlation Coefficient	,481	,261
		Sig. (2-tailed)	,113	,413
		N	12	12
	BAS_FUN	Correlation Coefficient	1,000	,644*
		Sig. (2-tailed)	.	,024
		N	12	12
	BAS_Reward	Correlation Coefficient	,644*	1,000
		Sig. (2-tailed)	,024	.
		N	12	12
	BIS	Correlation Coefficient	,529	,752**
		Sig. (2-tailed)	,077	,005
		N	12	12

Correlations[a]

			BIS
Spearman's rho	Stress-ACTH-max	Correlation Coefficient	,396
		Sig. (2-tailed)	,202
		N	12
	Stress-A-AUC	Correlation Coefficient	,266
		Sig. (2-tailed)	,404
		N	12
	Stress-C-max	Correlation Coefficient	,280
		Sig. (2-tailed)	,379
		N	12
	Stress-C-AUC	Correlation Coefficient	,149
		Sig. (2-tailed)	,645
		N	12
	Beck Depression Inventory	Correlation Coefficient	,101
		Sig. (2-tailed)	,754
		N	12
	SCL_90_tANX	Correlation Coefficient	,095
		Sig. (2-tailed)	,768
		N	12
	BAS_Drive	Correlation Coefficient	,083
		Sig. (2-tailed)	,798
		N	12
	BAS_FUN	Correlation Coefficient	,529
		Sig. (2-tailed)	,077
		N	12
	BAS_Reward	Correlation Coefficient	,752**
		Sig. (2-tailed)	,005
		N	12
	BIS	Correlation Coefficient	1,000
		Sig. (2-tailed)	.
		N	12

**. Correlation is significant at the 0.01 level (2-tailed).

*. Correlation is significant at the 0.05 level (2-tailed).

a. Status
(0=NC,1=ADI) = LC

Status

(0=NC,1=ADI) = OB

Correlations[a]

			Stress-ACTH-max	Stress-A-AUC
Spearman's rho	Stress-ACTH-max	Correlation Coefficient	1,000	,987**
		Sig. (2-tailed)	.	,000
		N	28	28
	Stress-A-AUC	Correlation Coefficient	,987**	1,000
		Sig. (2-tailed)	,000	.
		N	28	28
	Stress-C-max	Correlation Coefficient	,442*	,452*
		Sig. (2-tailed)	,019	,016
		N	28	28
	Stress-C-AUC	Correlation Coefficient	,446*	,462*
		Sig. (2-tailed)	,017	,013
		N	28	28
	Beck Depression Inventory	Correlation Coefficient	,164	,130
		Sig. (2-tailed)	,405	,509
		N	28	28
	SCL_90_tANX	Correlation Coefficient	,078	,064
		Sig. (2-tailed)	,744	,790
		N	20	20
	BAS_Drive	Correlation Coefficient	-,130	-,188
		Sig. (2-tailed)	,509	,337
		N	28	28
	BAS_FUN	Correlation Coefficient	-,176	-,182
		Sig. (2-tailed)	,370	,354
		N	28	28
	BAS_Reward	Correlation Coefficient	-,475*	-,486**
		Sig. (2-tailed)	,011	,009
		N	28	28
	BIS	Correlation Coefficient	-,236	-,247
		Sig. (2-tailed)	,227	,205
		N	28	28

Correlations[a]

			Stress-C-max	Stress-C-AUC
Spearman's rho	Stress-ACTH-max	Correlation Coefficient	,442*	,446*
		Sig. (2-tailed)	,019	,017
		N	28	28
	Stress-A-AUC	Correlation Coefficient	,452*	,462*
		Sig. (2-tailed)	,016	,013
		N	28	28
	Stress-C-max	Correlation Coefficient	1,000	,991**
		Sig. (2-tailed)	.	,000
		N	28	28
	Stress-C-AUC	Correlation Coefficient	,991**	1,000
		Sig. (2-tailed)	,000	.
		N	28	28
	Beck Depression Inventory	Correlation Coefficient	,170	,125
		Sig. (2-tailed)	,386	,527
		N	28	28
	SCL_90_tANX	Correlation Coefficient	-,107	-,089
		Sig. (2-tailed)	,653	,710
		N	20	20
	BAS_Drive	Correlation Coefficient	-,132	-,156
		Sig. (2-tailed)	,504	,429
		N	28	28
	BAS_FUN	Correlation Coefficient	-,036	-,070
		Sig. (2-tailed)	,857	,722
		N	28	28
	BAS_Reward	Correlation Coefficient	-,198	-,195
		Sig. (2-tailed)	,312	,319
		N	28	28
	BIS	Correlation Coefficient	-,349	-,349
		Sig. (2-tailed)	,069	,069
		N	28	28

Correlations[a]

			Beck Depression Inventory
Spearman's rho	Stress-ACTH-max	Correlation Coefficient	,164
		Sig. (2-tailed)	,405
		N	28
	Stress-A-AUC	Correlation Coefficient	,130
		Sig. (2-tailed)	,509
		N	28
	Stress-C-max	Correlation Coefficient	,170
		Sig. (2-tailed)	,386
		N	28
	Stress-C-AUC	Correlation Coefficient	,125
		Sig. (2-tailed)	,527
		N	28
	Beck Depression Inventory	Correlation Coefficient	1,000
		Sig. (2-tailed)	.
		N	28
	SCL_90_tANX	Correlation Coefficient	,360
		Sig. (2-tailed)	,119
		N	20
	BAS_Drive	Correlation Coefficient	-,101
		Sig. (2-tailed)	,609
		N	28
	BAS_FUN	Correlation Coefficient	-,339
		Sig. (2-tailed)	,077
		N	28
	BAS_Reward	Correlation Coefficient	-,309
		Sig. (2-tailed)	,109
		N	28
	BIS	Correlation Coefficient	,096
		Sig. (2-tailed)	,628
		N	28

Correlations[a]

			SCL_90_tANX	BAS_Drive
Spearman's rho	Stress-ACTH-max	Correlation Coefficient	,078	-,130
		Sig. (2-tailed)	,744	,509
		N	20	28
	Stress-A-AUC	Correlation Coefficient	,064	-,188
		Sig. (2-tailed)	,790	,337
		N	20	28
	Stress-C-max	Correlation Coefficient	-,107	-,132
		Sig. (2-tailed)	,653	,504
		N	20	28
	Stress-C-AUC	Correlation Coefficient	-,089	-,156
		Sig. (2-tailed)	,710	,429
		N	20	28
	Beck Depression Inventory	Correlation Coefficient	,360	-,101
		Sig. (2-tailed)	,119	,609
		N	20	28
	SCL_90_tANX	Correlation Coefficient	1,000	,146
		Sig. (2-tailed)	.	,538
		N	20	20
	BAS_Drive	Correlation Coefficient	,146	1,000
		Sig. (2-tailed)	,538	.
		N	20	28
	BAS_FUN	Correlation Coefficient	-,133	,528**
		Sig. (2-tailed)	,576	,004
		N	20	28
	BAS_Reward	Correlation Coefficient	,182	,403*
		Sig. (2-tailed)	,443	,034
		N	20	28
	BIS	Correlation Coefficient	,501*	,123
		Sig. (2-tailed)	,024	,533
		N	20	28

Correlations[a]

			BAS_FUN	BAS_Reward
Spearman's rho	Stress-ACTH-max	Correlation Coefficient	-,176	-,475*
		Sig. (2-tailed)	,370	,011
		N	28	28
	Stress-A-AUC	Correlation Coefficient	-,182	-,486**
		Sig. (2-tailed)	,354	,009
		N	28	28
	Stress-C-max	Correlation Coefficient	-,036	-,198
		Sig. (2-tailed)	,857	,312
		N	28	28
	Stress-C-AUC	Correlation Coefficient	-,070	-,195
		Sig. (2-tailed)	,722	,319
		N	28	28
	Beck Depression Inventory	Correlation Coefficient	-,339	-,309
		Sig. (2-tailed)	,077	,109
		N	28	28
	SCL_90_tANX	Correlation Coefficient	-,133	,182
		Sig. (2-tailed)	,576	,443
		N	20	20
	BAS_Drive	Correlation Coefficient	,528**	,403*
		Sig. (2-tailed)	,004	,034
		N	28	28
	BAS_FUN	Correlation Coefficient	1,000	,168
		Sig. (2-tailed)	.	,393
		N	28	28
	BAS_Reward	Correlation Coefficient	,168	1,000
		Sig. (2-tailed)	,393	.
		N	28	28
	BIS	Correlation Coefficient	,230	,305
		Sig. (2-tailed)	,239	,115
		N	28	28

Correlations[a]

			BIS
Spearman's rho	Stress-ACTH-max	Correlation Coefficient	-,236
		Sig. (2-tailed)	,227
		N	28
	Stress-A-AUC	Correlation Coefficient	-,247
		Sig. (2-tailed)	,205
		N	28
	Stress-C-max	Correlation Coefficient	-,349
		Sig. (2-tailed)	,069
		N	28
	Stress-C-AUC	Correlation Coefficient	-,349
		Sig. (2-tailed)	,069
		N	28
	Beck Depression Inventory	Correlation Coefficient	,096
		Sig. (2-tailed)	,628
		N	28
	SCL_90_tANX	Correlation Coefficient	,501*
		Sig. (2-tailed)	,024
		N	20
	BAS_Drive	Correlation Coefficient	,123
		Sig. (2-tailed)	,533
		N	28
	BAS_FUN	Correlation Coefficient	,230
		Sig. (2-tailed)	,239
		N	28
	BAS_Reward	Correlation Coefficient	,305
		Sig. (2-tailed)	,115
		N	28
	BIS	Correlation Coefficient	1,000
		Sig. (2-tailed)	.
		N	28

**. Correlation is significant at the 0.01 level (2-tailed).

*. Correlation is significant at the 0.05 level (2-tailed).

a. Status
(0=NC,1=ADI) = OB

```
NONPAR CORR
  /VARIABLES=SERT_BPND_Group_average CD BDI SCL_90_tANX BAS_Drive BAS_FUN B
AS_Reward BIS
  /PRINT=SPEARMAN TWOTAIL NOSIG
  /MISSING=PAIRWISE.
```

Nonparametric Correlations

Notes

Output Created		21-SEP-2022 00:01:23
Comments		
Input	Data	C:\Users\Christian Schinke\OneDrive - Charité - Universitätsmedizin Berlin\Promotion und Projekte Leipzig\02_DASB vs. HPA\06_Open_Data\01_Data_public.sav
	Active Dataset	DataSet1
	Filter	NOT INCLUSION_EXCLUSION =9 (FILTER)
	Weight	<none>
	Split File	Status (0=NC,1=ADI)
	N of Rows in Working Data File	40
Missing Value Handling	Definition of Missing	User-defined missing values are treated as missing.
	Cases Used	Statistics for each pair of variables are based on all the cases with valid data for that pair.
Syntax		NONPAR CORR<br>/VARIABLES=SERT_BPND_Group_average CD BDI SCL_90_tANX BAS_Drive BAS_FUN BAS_Reward BIS<br>/PRINT=SPEARMAN TWOTAIL NOSIG<br>/MISSING=PAIRWISE.

Notes

Resources	Processor Time	00:00:00,00
	Elapsed Time	00:00:00,01
	Number of Cases Allowed	285975 cases[a]

a. Based on availability of workspace memory

Status (0=NC,1=ADI) = LC

Correlations[a]

			VOI-BS_NUK_Group
Spearman's rho	VOI-BS_NUK_Group	Correlation Coefficient	1,000
		Sig. (2-tailed)	.
		N	12
	Head of the caudate	Correlation Coefficient	,448
		Sig. (2-tailed)	,145
		N	12
	Beck Depression Inventory	Correlation Coefficient	,313
		Sig. (2-tailed)	,322
		N	12
	SCL_90_tANX	Correlation Coefficient	-,065
		Sig. (2-tailed)	,841
		N	12
	BAS_Drive	Correlation Coefficient	-,019
		Sig. (2-tailed)	,953
		N	12
	BAS_FUN	Correlation Coefficient	-,219
		Sig. (2-tailed)	,494
		N	12
	BAS_Reward	Correlation Coefficient	-,468
		Sig. (2-tailed)	,125
		N	12
	BIS	Correlation Coefficient	-,336
		Sig. (2-tailed)	,285
		N	12

Correlations[a]

			Head of the caudate
Spearman's rho	VOI-BS_NUK_Group	Correlation Coefficient	,448
		Sig. (2-tailed)	,145
		N	12
	Head of the caudate	Correlation Coefficient	1,000
		Sig. (2-tailed)	.
		N	12
	Beck Depression Inventory	Correlation Coefficient	,238
		Sig. (2-tailed)	,457
		N	12
	SCL_90_tANX	Correlation Coefficient	-,485
		Sig. (2-tailed)	,110
		N	12
	BAS_Drive	Correlation Coefficient	,305
		Sig. (2-tailed)	,336
		N	12
	BAS_FUN	Correlation Coefficient	-,151
		Sig. (2-tailed)	,640
		N	12
	BAS_Reward	Correlation Coefficient	-,350
		Sig. (2-tailed)	,265
		N	12
	BIS	Correlation Coefficient	,011
		Sig. (2-tailed)	,974
		N	12

Correlations[a]

			Beck Depression Inventory
Spearman's rho	VOI-BS_NUK_Group	Correlation Coefficient	,313
		Sig. (2-tailed)	,322
		N	12
	Head of the caudate	Correlation Coefficient	,238
		Sig. (2-tailed)	,457
		N	12
	Beck Depression Inventory	Correlation Coefficient	1,000
		Sig. (2-tailed)	.
		N	12
	SCL_90_tANX	Correlation Coefficient	,149
		Sig. (2-tailed)	,644
		N	12
	BAS_Drive	Correlation Coefficient	-,170
		Sig. (2-tailed)	,597
		N	12
	BAS_FUN	Correlation Coefficient	-,096
		Sig. (2-tailed)	,766
		N	12
	BAS_Reward	Correlation Coefficient	-,275
		Sig. (2-tailed)	,387
		N	12
	BIS	Correlation Coefficient	,101
		Sig. (2-tailed)	,754
		N	12

Correlations[a]

			SCL_90_tANX	BAS_Drive
Spearman's rho	VOI-BS_NUK_Group	Correlation Coefficient	-,065	-,019
		Sig. (2-tailed)	,841	,953
		N	12	12
	Head of the caudate	Correlation Coefficient	-,485	,305
		Sig. (2-tailed)	,110	,336
		N	12	12
	Beck Depression Inventory	Correlation Coefficient	,149	-,170
		Sig. (2-tailed)	,644	,597
		N	12	12
	SCL_90_tANX	Correlation Coefficient	1,000	-,280
		Sig. (2-tailed)	.	,378
		N	12	12
	BAS_Drive	Correlation Coefficient	-,280	1,000
		Sig. (2-tailed)	,378	.
		N	12	12
	BAS_FUN	Correlation Coefficient	-,028	,481
		Sig. (2-tailed)	,931	,113
		N	12	12
	BAS_Reward	Correlation Coefficient	,100	,261
		Sig. (2-tailed)	,758	,413
		N	12	12
	BIS	Correlation Coefficient	,095	,083
		Sig. (2-tailed)	,768	,798
		N	12	12

Correlations[a]

			BAS_FUN	BAS_Reward
Spearman's rho	VOI-BS_NUK_Group	Correlation Coefficient	-,219	-,468
		Sig. (2-tailed)	,494	,125
		N	12	12
	Head of the caudate	Correlation Coefficient	-,151	-,350
		Sig. (2-tailed)	,640	,265
		N	12	12
	Beck Depression Inventory	Correlation Coefficient	-,096	-,275
		Sig. (2-tailed)	,766	,387
		N	12	12
	SCL_90_tANX	Correlation Coefficient	-,028	,100
		Sig. (2-tailed)	,931	,758
		N	12	12
	BAS_Drive	Correlation Coefficient	,481	,261
		Sig. (2-tailed)	,113	,413
		N	12	12
	BAS_FUN	Correlation Coefficient	1,000	,644*
		Sig. (2-tailed)	.	,024
		N	12	12
	BAS_Reward	Correlation Coefficient	,644*	1,000
		Sig. (2-tailed)	,024	.
		N	12	12
	BIS	Correlation Coefficient	,529	,752**
		Sig. (2-tailed)	,077	,005
		N	12	12

Correlations[a]

			BIS
Spearman's rho	VOI-BS_NUK_Group	Correlation Coefficient	-,336
		Sig. (2-tailed)	,285
		N	12
	Head of the caudate	Correlation Coefficient	,011
		Sig. (2-tailed)	,974
		N	12
	Beck Depression Inventory	Correlation Coefficient	,101
		Sig. (2-tailed)	,754
		N	12
	SCL_90_tANX	Correlation Coefficient	,095
		Sig. (2-tailed)	,768
		N	12
	BAS_Drive	Correlation Coefficient	,083
		Sig. (2-tailed)	,798
		N	12
	BAS_FUN	Correlation Coefficient	,529
		Sig. (2-tailed)	,077
		N	12
	BAS_Reward	Correlation Coefficient	,752**
		Sig. (2-tailed)	,005
		N	12
	BIS	Correlation Coefficient	1,000
		Sig. (2-tailed)	.
		N	12

*. Correlation is significant at the 0.05 level (2-tailed).

**. Correlation is significant at the 0.01 level (2-tailed).

a. Status
(0=NC,1=ADI) = LC

Status
(0=NC,1=ADI) = OB

Correlations[a]

			VOI-BS_NUK_Group
Spearman's rho	VOI-BS_NUK_Group	Correlation Coefficient	1,000
		Sig. (2-tailed)	.
		N	28
	Head of the caudate	Correlation Coefficient	,856**
		Sig. (2-tailed)	,000
		N	28
	Beck Depression Inventory	Correlation Coefficient	,343
		Sig. (2-tailed)	,074
		N	28
	SCL_90_tANX	Correlation Coefficient	-,294
		Sig. (2-tailed)	,209
		N	20
	BAS_Drive	Correlation Coefficient	-,257
		Sig. (2-tailed)	,187
		N	28
	BAS_FUN	Correlation Coefficient	-,189
		Sig. (2-tailed)	,336
		N	28
	BAS_Reward	Correlation Coefficient	-,565**
		Sig. (2-tailed)	,002
		N	28
	BIS	Correlation Coefficient	-,221
		Sig. (2-tailed)	,259
		N	28

Correlations[a]

			Head of the caudate
Spearman's rho	VOI-BS_NUK_Group	Correlation Coefficient	,856**
		Sig. (2-tailed)	,000
		N	28
	Head of the caudate	Correlation Coefficient	1,000
		Sig. (2-tailed)	.
		N	28
	Beck Depression Inventory	Correlation Coefficient	,313
		Sig. (2-tailed)	,105
		N	28
	SCL_90_tANX	Correlation Coefficient	-,213
		Sig. (2-tailed)	,366
		N	20
	BAS_Drive	Correlation Coefficient	-,110
		Sig. (2-tailed)	,579
		N	28
	BAS_FUN	Correlation Coefficient	-,153
		Sig. (2-tailed)	,437
		N	28
	BAS_Reward	Correlation Coefficient	-,579**
		Sig. (2-tailed)	,001
		N	28
	BIS	Correlation Coefficient	-,264
		Sig. (2-tailed)	,175
		N	28

Correlations[a]

			Beck Depression Inventory
Spearman's rho	VOI-BS_NUK_Group	Correlation Coefficient	,343
		Sig. (2-tailed)	,074
		N	28
	Head of the caudate	Correlation Coefficient	,313
		Sig. (2-tailed)	,105
		N	28
	Beck Depression Inventory	Correlation Coefficient	1,000
		Sig. (2-tailed)	.
		N	28
	SCL_90_tANX	Correlation Coefficient	,360
		Sig. (2-tailed)	,119
		N	20
	BAS_Drive	Correlation Coefficient	-,101
		Sig. (2-tailed)	,609
		N	28
	BAS_FUN	Correlation Coefficient	-,339
		Sig. (2-tailed)	,077
		N	28
	BAS_Reward	Correlation Coefficient	-,309
		Sig. (2-tailed)	,109
		N	28
	BIS	Correlation Coefficient	,096
		Sig. (2-tailed)	,628
		N	28

Correlations[a]

			SCL_90_tANX	BAS_Drive
Spearman's rho	VOI-BS_NUK_Group	Correlation Coefficient	-,294	-,257
		Sig. (2-tailed)	,209	,187
		N	20	28
	Head of the caudate	Correlation Coefficient	-,213	-,110
		Sig. (2-tailed)	,366	,579
		N	20	28
	Beck Depression Inventory	Correlation Coefficient	,360	-,101
		Sig. (2-tailed)	,119	,609
		N	20	28
	SCL_90_tANX	Correlation Coefficient	1,000	,146
		Sig. (2-tailed)	.	,538
		N	20	20
	BAS_Drive	Correlation Coefficient	,146	1,000
		Sig. (2-tailed)	,538	.
		N	20	28
	BAS_FUN	Correlation Coefficient	-,133	,528**
		Sig. (2-tailed)	,576	,004
		N	20	28
	BAS_Reward	Correlation Coefficient	,182	,403*
		Sig. (2-tailed)	,443	,034
		N	20	28
	BIS	Correlation Coefficient	,501*	,123
		Sig. (2-tailed)	,024	,533
		N	20	28

Correlations[a]

			BAS_FUN	BAS_Reward
Spearman's rho	VOI-BS_NUK_Group	Correlation Coefficient	-,189	-,565**
		Sig. (2-tailed)	,336	,002
		N	28	28
	Head of the caudate	Correlation Coefficient	-,153	-,579**
		Sig. (2-tailed)	,437	,001
		N	28	28
	Beck Depression Inventory	Correlation Coefficient	-,339	-,309
		Sig. (2-tailed)	,077	,109
		N	28	28
	SCL_90_tANX	Correlation Coefficient	-,133	,182
		Sig. (2-tailed)	,576	,443
		N	20	20
	BAS_Drive	Correlation Coefficient	,528**	,403*
		Sig. (2-tailed)	,004	,034
		N	28	28
	BAS_FUN	Correlation Coefficient	1,000	,168
		Sig. (2-tailed)	.	,393
		N	28	28
	BAS_Reward	Correlation Coefficient	,168	1,000
		Sig. (2-tailed)	,393	.
		N	28	28
	BIS	Correlation Coefficient	,230	,305
		Sig. (2-tailed)	,239	,115
		N	28	28

Correlations[a]

			BIS
Spearman's rho	VOI-BS_NUK_Group	Correlation Coefficient	-,221
		Sig. (2-tailed)	,259
		N	28
	Head of the caudate	Correlation Coefficient	-,264
		Sig. (2-tailed)	,175
		N	28
	Beck Depression Inventory	Correlation Coefficient	,096
		Sig. (2-tailed)	,628
		N	28
	SCL_90_tANX	Correlation Coefficient	,501*
		Sig. (2-tailed)	,024
		N	20
	BAS_Drive	Correlation Coefficient	,123
		Sig. (2-tailed)	,533
		N	28
	BAS_FUN	Correlation Coefficient	,230
		Sig. (2-tailed)	,239
		N	28
	BAS_Reward	Correlation Coefficient	,305
		Sig. (2-tailed)	,115
		N	28
	BIS	Correlation Coefficient	1,000
		Sig. (2-tailed)	.
		N	28

**. Correlation is significant at the 0.01 level (2-tailed).

*. Correlation is significant at the 0.05 level (2-tailed).

a. Status
(0=NC,1=ADI) = OB

```
 SORT CASES  BY Participant_ID
SPLIT FILE SEPARATE BY Participant_ID.

>Error # 701 in column 1.  Text: SPLIT
>An undefined variable name, or a scratch or system variable was specified
in a
```

```
>variable list which accepts only standard variables.  Check spelling and
>verify the existence of this variable.
>Execution of this command stops.

USE ALL.
COMPUTE filter_$=(NOT INCLUSION_EXCLUSION=9).
VARIABLE LABELS filter_$ 'NOT INCLUSION_EXCLUSION=9 (FILTER)'.
VALUE LABELS filter_$ 0 'Not Selected' 1 'Selected'.
FORMATS filter_$ (f1.0).
FILTER BY filter_$.
EXECUTE.

SORT CASES  BY Status_0_NO_1_OB.
SPLIT FILE LAYERED BY Status_0_NO_1_OB.
DATASET ACTIVATE DataSet1.
NONPAR CORR
  /VARIABLES=RAPHE ACTH_MAX ACTH_AUC CRT_MAX CRT_AUC
  /PRINT=SPEARMAN TWOTAIL NOSIG
  /MISSING=PAIRWISE.
```

Nonparametric Correlations

Notes

Output Created		21-SEP-2022 11:19:44
Comments		
Input	Data	C:\Users\Christian Schinke\OneDrive - Charité - Universitätsmedizin Berlin\Promotion und Projekte Leipzig\02_DASB vs. HPA\06_Open_Data\01_Data_public.sav
	Active Dataset	DataSet1
	Filter	NOT INCLUSION_EXCLUSION=9 (FILTER)
	Weight	<none>
	Split File	Status (0=NC,1=ADI)
	N of Rows in Working Data File	40

Notes

Missing Value Handling	Definition of Missing	User-defined missing values are treated as missing.
	Cases Used	Statistics for each pair of variables are based on all the cases with valid data for that pair.
Syntax		NONPAR CORR /VARIABLES=RAPHE ACTH_MAX ACTH_AUC CRT_MAX CRT_AUC /PRINT=SPEARMAN TWOTAIL NOSIG /MISSING=PAIRWISE.
Resources	Processor Time	00:00:00,00
	Elapsed Time	00:00:00,00
	Number of Cases Allowed	393216 cases[a]

a. Based on availability of workspace memory

```
[DataSet1] C:\Users\Christian Schinke\OneDrive - Charité - Universitätsmedi
zin Berlin\Promotion und Projekte Leipzig\02_DASB vs. HPA\06_Open_Data\01_D
ata_public.sav
```

Correlations

			Status (0=NC,1=ADI)	
			NO	
			Raphe	Stress-ACTH-max
Spearman's rho	Raphe	Correlation Coefficient	1,000	,063
		Sig. (2-tailed)	.	,846
		N	12	12
	Stress-ACTH-max	Correlation Coefficient	,063	1,000
		Sig. (2-tailed)	,846	.
		N	12	12
	Stress-A-AUC	Correlation Coefficient	,028	,944**
		Sig. (2-tailed)	,931	,000
		N	12	12
	Stress-C-max	Correlation Coefficient	-,042	,566
		Sig. (2-tailed)	,897	,055
		N	12	12
	Stress-C-AUC	Correlation Coefficient	,140	,413
		Sig. (2-tailed)	,665	,183
		N	12	12

Correlations

			Status (0=NC,1=ADI) NO	
			Stress-A-AUC	Stress-C-max
Spearman's rho	Raphe	Correlation Coefficient	,028	-,042
		Sig. (2-tailed)	,931	,897
		N	12	12
	Stress-ACTH-max	Correlation Coefficient	,944**	,566
		Sig. (2-tailed)	,000	,055
		N	12	12
	Stress-A-AUC	Correlation Coefficient	1,000	,524
		Sig. (2-tailed)	.	,080
		N	12	12
	Stress-C-max	Correlation Coefficient	,524	1,000
		Sig. (2-tailed)	,080	.
		N	12	12
	Stress-C-AUC	Correlation Coefficient	,336	,916**
		Sig. (2-tailed)	,286	,000
		N	12	12

Correlations

			Status (0=NC,1=ADI)	
			NO	OB
			Stress-C-AUC	Raphe
Spearman's rho	Raphe	Correlation Coefficient	,140	1,000
		Sig. (2-tailed)	,665	.
		N	12	28
	Stress-ACTH-max	Correlation Coefficient	,413	-,076
		Sig. (2-tailed)	,183	,700
		N	12	28
	Stress-A-AUC	Correlation Coefficient	,336	-,072
		Sig. (2-tailed)	,286	,717
		N	12	28
	Stress-C-max	Correlation Coefficient	,916**	-,020
		Sig. (2-tailed)	,000	,921
		N	12	28
	Stress-C-AUC	Correlation Coefficient	1,000	-,076
		Sig. (2-tailed)	.	,702
		N	12	28

Correlations

			Status (0=NC,1=ADI)	
			OB	
			Stress-ACTH-max	Stress-A-AUC
Spearman's rho	Raphe	Correlation Coefficient	-,076	-,072
		Sig. (2-tailed)	,700	,717
		N	28	28
	Stress-ACTH-max	Correlation Coefficient	1,000	,987**
		Sig. (2-tailed)	.	,000
		N	28	28
	Stress-A-AUC	Correlation Coefficient	,987**	1,000
		Sig. (2-tailed)	,000	.
		N	28	28
	Stress-C-max	Correlation Coefficient	,442*	,452*
		Sig. (2-tailed)	,019	,016
		N	28	28
	Stress-C-AUC	Correlation Coefficient	,446*	,462*
		Sig. (2-tailed)	,017	,013
		N	28	28

Correlations

			Status (0=NC,1=ADI) OB Stress-C-max	Status (0=NC,1=ADI) OB Stress-C-AUC
Spearman's rho	Raphe	Correlation Coefficient	-,020	-,076
		Sig. (2-tailed)	,921	,702
		N	28	28
	Stress-ACTH-max	Correlation Coefficient	,442*	,446*
		Sig. (2-tailed)	,019	,017
		N	28	28
	Stress-A-AUC	Correlation Coefficient	,452*	,462*
		Sig. (2-tailed)	,016	,013
		N	28	28
	Stress-C-max	Correlation Coefficient	1,000	,991**
		Sig. (2-tailed)	.	,000
		N	28	28
	Stress-C-AUC	Correlation Coefficient	,991**	1,000
		Sig. (2-tailed)	,000	.
		N	28	28

**. Correlation is significant at the 0.01 level (2-tailed).

*. Correlation is significant at the 0.05 level (2-tailed).
